# Use of Large Language Models to Classify Epidemiological Characteristics in Synthetic and Real-World Social Media Posts About Conjunctivitis Outbreaks: Infodemiology Study

**DOI:** 10.2196/65226

**Published:** 2025-07-02

**Authors:** Michael S Deiner, Russell Y Deiner, Cherie Fathy, Natalie A Deiner, Vagelis Hristidis, Stephen D McLeod, Thomas J Bukowski, Thuy Doan, Gerami D Seitzman, Thomas M Lietman, Travis C Porco

**Affiliations:** 1 Francis I Proctor Foundation for Research in Ophthalmology, University of California, San Francisco San Francisco, CA United States; 2 Department of Ophthalmology, University of California, San Francisco San Francisco, CA United States; 3 Abraham Lincoln High School, San Francisco, CA San Francisco, CA United States; 4 Document Control Center, Food and Drug Administration, Center for Biologics Evaluation and Research Silver Spring, CA United States; 5 College of Letters and Sciences University of California, Santa Barbara Santa Barbara, CA United States; 6 Department of Computer Science and Engineering University of California, Riverside Riverside, CA United States; 7 American Academy of Ophthalmology San Francisco, CA United States; 8 Department of Epidemiology and Biostatistics, University of California, San Francisco San Francisco, CA United States

**Keywords:** conjunctivitis, microblog, social media, generative pretrained transformers, epidemic classification, forums, Twitter/X, YouTube, infectious eye disease, large language models, infodemiology, ocular, outbreak, epidemic

## Abstract

**Background:**

The use of web-based search and social media can help identify epidemics, potentially earlier than clinical methods or even potentially identifying unreported outbreaks. Monitoring for eye-related epidemics, such as conjunctivitis outbreaks, can facilitate early public health intervention to reduce transmission and ocular comorbidities. However, monitoring social media content for conjunctivitis outbreaks is costly and laborious. Large language models (LLMs) could overcome these barriers by assessing the likelihood that real-world outbreaks are being described. However, public health actions for likely outbreaks could benefit more by knowing additional epidemiological characteristics, such as outbreak type, size, and severity.

**Objective:**

We aimed to assess whether and how well LLMs can classify epidemiological features from social media posts beyond conjunctivitis outbreak probability, including outbreak type, size, severity, etiology, and community setting. We used a validation framework comparing LLM classifications to those of other LLMs and human experts.

**Methods:**

We wrote code to generate synthetic conjunctivitis outbreak social media posts, embedded with specific preclassified epidemiological features to simulate various infectious eye disease outbreak and control scenarios. We used these posts to develop effective LLM prompts and test the capabilities of multiple LLMs. For top-performing LLMs, we gauged their practical utility in real-world epidemiological surveillance by comparing their assessments of Twitter/X, forum, and YouTube conjunctivitis posts. Finally, human raters also classified the posts, and we compared their classifications to those of a leading LLM for validation. Comparisons entailed correlation or sensitivity and specificity statistics.

**Results:**

We assessed 7 LLMs for effectively classifying epidemiological data from 1152 synthetic posts, 370 Twitter/X posts, 290 forum posts, and 956 YouTube posts. Despite some discrepancies, the LLMs demonstrated a reliable capacity for nuanced epidemiological analysis across various data sources and compared to humans or between LLMs. Notably, GPT-4 and Mixtral 8x22b exhibited high performance, predicting conjunctivitis outbreak characteristics such as probability (GPT-4: correlation=0.73), size (Mixtral 8x22b: correlation=0.82), and type (infectious, allergic, or environmentally caused); however, there were notable exceptions. Assessing synthetic and real-world posts for etiological factors, infectious eye disease specialist validations revealed that GPT-4 had high specificity (0.83-1.00) but variable sensitivity (0.32-0.71). Interrater reliability analyses showed that LLM-expert agreement exceeded expert-expert agreement for severity assessment (intraclass correlation coefficient=0.69 vs 0.38), while agreement varied by condition type (κ=0.37-0.94).

**Conclusions:**

This investigation into the potential of LLMs for public health infoveillance suggests effectiveness in classifying key epidemiological characteristics from social media content about conjunctivitis outbreaks. Future studies should further explore LLMs’ potential to support public health monitoring through the automated assessment and classification of potential infectious eye disease or other outbreaks. Their optimal role may be to act as a first line of documentation, alerting public health organizations for the follow-up of LLM-detected and -classified small, early outbreaks, with a focus on the most severe ones.

## Introduction

### Social Media in Epidemic Detection

Traditional clinical data reporting and surveillance systems monitoring for conjunctivitis outbreaks can allow for early public health intervention, which has been shown to reduce the overall societal impact [1,2]. Surveillance can also act as an early indicator of systemic diseases, including COVID-19 [3-6] or vision-threatening effects of contaminated eye care products [7]. However, in many countries, including in the United States, such traditional reporting and surveillance systems do not exist. Alternative approaches to monitor for outbreaks outside of clinical reporting may therefore be beneficial in identifying and alerting for potential outbreaks early enough to enable public health action.

Social media platforms have become an increasingly important source of real-world health information; for example, a recent study found that approximately 1 in 5 American adults surveyed shares personal health information on social media, and more than a quarter engage with others about similar health challenges [8]. Moreover, nearly 5 billion people worldwide use social media platforms such as Twitter/X, Facebook, YouTube, Instagram, and TikTok [9-11]. These sources likely generate thousands to tens of thousands or more posts each month related to health issues [12,13], including ocular conditions [14-18], representing a valuable but largely untapped resource for public eye health surveillance and research. The use of web-based search and social media in health research to detect epidemics or outbreaks could potentially allow the identification of epidemics more quickly than clinical surveillance or the detection of epidemics that might otherwise have gone unreported [14-16,18-45]. Ocular conditions, particularly conjunctivitis, are frequently discussed on social media platforms [14-18], presumably due to their visible nature, contagious potential, and burden on daily life [46]. Timeseries counts of social media posts about conjunctivitis can be used for timeseries-based detection of outbreaks [14,25,28]. Another approach, identifying outbreaks based on the content of social media posts, has the potential to further improve monitoring and detection for outbreaks across many health conditions, including conjunctivitis, but conducting such analyses using social media posts requires natural language understanding of social media content and has been limited by the costs of using human raters or alternatively by the insufficiency of computational natural language processing [39,40,44,45,47-51]. More recently, large language models (LLMs) have been explored as an alternative tool for this purpose, but thus far little has been fully studied related to eye disease [18,45,51-53].

### LLMs for Classification of Outbreak Features

In our prior LLM study [18], we had investigated the potential of an LLM to determine whether conjunctivitis outbreaks might be detected based on the content of posts about the condition on Twitter (subsequently rebranded as X). We had examined whether GPT-3.5 and GPT-4 can provide probabilistic assessments of whether these posts about conjunctivitis could indicate an outbreak. Mean probabilistic assessments from GPT-4 in the published study correlated well with human raters (*r=*0.60, 95% CI 0.47-0.70) [18]. The mean of these elicited percentages from the posts about conjunctivitis correlated with both post volume and the occurrence of some known epidemics. We did not assess the ability of LLMs to assess the probability of epidemics based on content sourced from other forms of social media, such as web-based forums or blogs. In addition, we observed that quantitative analysis of post clusters for timeseries of counts over time can be insufficient because this approach requires a minimum volume of posts for detecting differences in the counts of posts over time. It is possible that, in some instances, this threshold may never be met.

Our prior study led us to recognize that individual social media posts can contain valuable signals about infectious eye outbreaks, including detailed features about very small or early outbreaks not yet detectable through traditional surveillance methods, such as analyses of timeseries counts per day, clinical data, or other means [18]. While such posts could provide important early warning signals, any vision of future implementation of a system requiring regular, ongoing real-time human review of all potential outbreak–related posts was clearly not feasible. This unmet need—the gap between valuable social media signals and limited human resources for monitoring—motivated our investigation into LLMs’ potential to automate the content assessment process. We hypothesized that LLMs could extract reliable information from posts not only about the probability of epidemics but also about specific characteristics (such as size, severity, or etiology) of suspected conjunctivitis epidemics. If so, an automated approach could potentially enable LLM-based continuous surveillance and characterization of vast amounts of application programming interface (API)–harvested conjunctivitis-keyword–based social media content about infectious eye disease, red flagging only the most concerning posts for human review. Such a system could potentially identify concerning outbreak signals before clinical cases emerge or before sufficient numbers of posts are available for traditional timeseries analysis, particularly for initial posts about early small but severe epidemics that might otherwise go undetected if monitoring is occurring only for increases in timeseries counts [14,18,28]. If successful, public health human expertise could then be focused where it matters most—investigating automatically detected signals of potentially serious outbreaks requiring intervention.

The aim of this infoveillance study was therefore to address the following research question: in addition to assessing the probability of an outbreak, can an LLM classify key epidemiological conjunctivitis outbreak characteristics (outbreak type and size, severity and etiology of cases, and other health conditions mentioned) from individual social media posts originating from a number of social media platforms? We tested the general hypothesis that the correlation, sensitivity, and specificity results would be better for some LLMs than for others and be better for some of the simpler features being assessed (eg, outbreak probability or type) than for harder-to-discern features (eg, severity or etiology). Using a diverse set of 7 LLMs to conduct several tiers of validations, we assessed whether LLMs can provide probabilistic assessments of the chance that each post corresponds to an outbreak as well as classify other characteristics. We implemented a validation framework using synthetic data with predefined characteristics that act as a gold standard (refer to the Methods section), allowing us to efficiently assess LLM performance. We then used comparative validation between LLMs and human validation by domain experts to further strengthen the reliability of our findings, using real-world posts from multiple social media platforms. If successful, leveraging these classifications could enable us to study the nature of outbreaks, including those that may never be present, detected, or well characterized in clinical datasets. Moreover, this approach has the potential for identifying higher-risk severe epidemics, which in turn could aid public health authorities in taking timely action earlier, specifically targeted to address severe outbreaks.

## Methods

### Overview

LLMs were used to extract and analyze public health information from social media posts. Our analysis focused on classifying several key outbreak characteristics: outbreak probability (essential for filtering noise and identifying genuine outbreak signals in social media data), outbreak type (necessary for tailoring appropriate interventions and response strategies), outbreak size (critical for assessing the scale of response needed and resource allocation), outbreak severity (crucial for triaging public health responses and identifying outbreaks requiring immediate intervention), outbreak etiology (important for determining appropriate treatment strategies and containment measures), the presence of other health conditions (valuable for understanding potential complications and identifying secondary health impacts), and the community setting and location (vital for mapping outbreak spread and coordinating localized responses; Textbox 1). These characteristics were chosen to provide a comprehensive epidemiological profile of potential outbreaks based on social media content. First, for efficient model development and to rigorously evaluate LLM capabilities, we developed a framework for generating synthetic social media posts with predefined epidemiological characteristics, using templates, controlled vocabularies, and random sampling. This allowed the generation of diverse posts simulating various preclassified outbreak scenarios, health conditions, and demographics, spanning a wide range of conditions, including rare ones, for LLM assessment. From an initial pool of multiple different LLMs, we identified top performers based on their performance on these synthetic datasets after fine-tuning prompts for specific tasks. We then assessed LLM performance through a combination of correlation analysis, sensitivity and specificity calculations, and human validation by domain experts by using a multistage evaluation process to assess LLM performance in the following order: (1) correlation analysis between LLM outputs and known values for synthetic posts, (2) sensitivity and specificity calculations for outbreak type and health condition classification, (3) intermodel comparisons of LLMs, and (4) human validation by both nonexpert raters and domain experts. In this multilayered approach, we used synthetic posts for the systematic evaluation of LLM performance and then conducted an essential validation of LLM classifications of real-world posts through both cross-LLM comparisons and human expert review. This allowed us to leverage the advantages of synthetic data while assessing real-world applicability.

**Key conjunctivitis outbreak epidemiological features in social media posts classified by large language models (LLMs) in this study. To ensure clarity and consistency throughout our analysis, we identified 7 key epidemiological features that we aimed to extract from social media posts for evaluation. These features formed the core of our assessment criteria for both LLM performance and human validation.**

- Outbreak probability: the likelihood that the post describes a genuine outbreak of conjunctivitis (0%-100%)
- Outbreak type: the classification of the outbreak as infectious, allergic, environmentally caused, or acute hemorrhagic conjunctivitis
- Outbreak size (number of cases): the estimated number of individuals affected by the outbreak
- Outbreak severity: the impact on health, categorized as mild, moderate, strong, or severe
- Etiology: the cause of the outbreak, if infectious (eg, viral, bacterial, acute hemorrhagic conjunctivitis, COVID-19, or unspecified)
- Other health conditions: any additional health conditions mentioned in the post beyond conjunctivitis
- Community setting: the geolocation (city, state, etc) or setting (school, dormitory, or house) of the outbreak

### Data Collection and Preparation

#### Synthetic Post Generation

To test the ability of an LLM to identify outbreak characteristics across a wide range of characteristics (eg, affecting a single household vs a state, mild cases vs severe cases, infectious vs allergic or environmentally caused, systemic vs only ocular, and bacterial vs viral), we must first have a set of posts that represent this diversity. Posts about the potentially more important outbreaks, such as those causing acute hemorrhagic conjunctivitis (AHC), may appear rarely in social media due to their rarity. However, their public health significance, given their potential for rapid spread and severity, demands reliable detection capabilities. To ensure that our LLM-based approach for classifying conjunctivitis outbreaks can properly characterize such clinically important but rare events, we used an established solution in the field: we developed software code to generate preclassified synthetic posts to represent a full range of characteristics [54-58]. Regarding AHC, our synthetic posts were designed to mainly describe the clinical presentation and symptoms of AHC (eg, general terms for bleeding such as “blood” and “hemorrhage”), rather than using specific terminology such as “AHC” or “acute hemorrhagic.” Thus, our structured generation of synthetic posts allowed us to generate a sufficient number of posts about AHC (and other rare conditions) to enable us to statistically validate whether the LLMs were able to identify these rare conditions based on symptom descriptions as they might naturally appear in real-world social media content.

Preclassified hard-coded epidemiological classification values (eg, a post with a known conjunctivitis health severity level, such as “mild” or “severe”) for each generated post also provide an efficient way to optimize prompts and test for sensitivity and specificity. This approach bypasses the traditionally time-consuming and costly process of having humans assign classification scores to posts obtained from social media platforms. Other advantages of creating and using synthetic social media posts include the ability to generate large quantities of data at very low cost, which supports statistical power; full control over variables, allowing for greater certainty in experiments; the flexibility to modify content during iterative model development and refinement; and complete protection of privacy [57,58]. For all these reasons, we developed code to create synthetic posts, which allowed us to design prompts that could guide the LLMs to correctly classify these preclassified posts before we proceeded to validation with real-world posts or human subject matter experts.

Our modular hard-coded system allowed us to generate synthetic posts in which the classifications for all our epidemiological characteristics of interest were preassigned (and did not need to be extracted manually) for planned comparisons to the values of these same parameters extracted from LLMs. This allowed for rapid assessment of any given LLM platform’s ability to extract information of interest from these synthetic posts, comparing values elicited by the LLMs to values preassigned by our hard-coded system to each post for all parameters of interest in the posts. Our synthetic posts were constructed by combining components from predefined categories, specifically outbreak probability indicators, outbreak settings, disease severity descriptors, causative organisms, and associated symptoms. We selected these at random and concatenated components from each category based on specified probabilities to generate plausible posts. Disease severity was defined as the maximum severity among the components within a post. Nonepidemic phrases and noninfectious forms, such as environmental causes, were incorporated probabilistically to ensure a diverse dataset with controls. Each synthetic post was generated and output in CSV format, with additional columns containing the preclassified values for each outbreak characteristic of that post. Examples of 4 generated synthetic posts are provided in the Results section. We generated 1152 synthetic posts using the algorithm, which was implemented in R (version 4.3 for MacIntosh; R Foundation for Statistical Computing). To be clear, the generation of these synthetic posts and their preassigned classifications was not carried out using LLMs. The code (algorithm) for our computational framework for generating synthetic posts mimicking outbreak-related social media content through a structured probabilistic approach is included in Multimedia Appendix 1. A deeper dive into our methods for synthetic post generation and bias mitigation is also provided in Multimedia Appendix 1.

#### Twitter/X, Forum, and YouTube Posts

We collected posts from Twitter/X, web-based forums, and YouTube using the Brandwatch interface, a commercially available social media listening tool that retrieves social media posts and other web-based content based on user-defined queries [18]. For Twitter/X and forum posts, we used a Boolean query containing words in multiple languages representing conjunctivitis (eg, “conjuctivitis,” “conjuntivitis,” “conjuntivite,” and “pink eye”), tailored to enrich for posts about outbreaks where cases and symptoms were described, while excluding irrelevant content such as that related to animals, artistic references, obscenities, or celebrities having conjunctivitis. For YouTube, we used the same core multilanguage conjunctivitis-related terms but with some modifications allowing for a broader query: additional terms (eg, “eye infection,” hashtag variations, and wildcard patterns) and different proximity requirements for outbreak-related terms. The data collection window for Twitter/X began on October 16, 2020, and for forums on October 16, 2018; both sources were sampled through October 16, 2023, resulting in 370 Twitter/X posts and 290 forum posts. The YouTube data collection spanned March 2013 to March 2025; from 43,995 YouTube posts containing conjunctivitis-related terms, we created a balanced dataset of 956 (2.17%) posts by combining all 478 (50%) posts containing explicit outbreak or spread-related terms with a random sample of 478 (50%) posts without such terms. The complete Boolean query details for all platforms are provided in Multimedia Appendix 2.

### LLM Selection and Implementation

Our selection of LLMs for this study was based on several key criteria:

1. Performance benchmarks: we prioritized models that have demonstrated strong performance in general language understanding tasks, as indicated by their scores on benchmarks and leaderboards such as Large Model Systems Organization and Vectara.
2. Availability and accessibility: we chose models that were publicly available or accessible through well-documented APIs, ensuring the reproducibility of our study.
3. Prevalence in the literature: we included models that are more commonly used and cited in published research.
4. Model size and capability: we included a range of model sizes to investigate the relationship between model scale and performance in our specific task.
5. Diversity of training approaches: we selected models from different organizations to capture a variety of training methodologies and data sources.
6. Recency: we focused on models that were state of the art at the time of our study to ensure the relevance of our findings.

On the basis of these criteria, we selected the following LLMs: GPT-3.5 (gpt-3.5-turbo-0613), GPT-4 (gpt-4-0314), and GPT-4o (gpt-4o-2024-05-13; OpenAI); Claude 3 Sonnet Claude 3 Opus (Anthropic); Mixtral 8x22b (Mistral AI); and LlaMa 3 70B (Meta AI). GPT-3.5 and GPT-4 were chosen for their strong general performance and widespread use in research; GPT-4o allowed comparing performance with the standard GPT-4 model; Claude 3 Sonnet was chosen as an alternative large-scale model, with a different training approach than GPT; Mixtral 8x22b was a recent, alternative open-weight model with strong performance; and LlaMa 3 70B, a large, open-source model, was selected to represent academic research efforts. This selection therefore provided a representative sample of high-performing models with diverse characteristics, allowing for a comprehensive evaluation of LLM capabilities in epidemiological analysis of social media data.

To send prompts and receive responses, we used vendor-provided APIs for OpenAI and Anthropic. We conducted inference for Mixtral 8x22b and LlaMa 3 70B on the Octo Labs platform (Octo Labs). A temperature setting of 0.0 was used for each inference.

### Iterative Prompt Optimization

We used synthetic post sets—typically containing ≤250 posts—for iterative prompt development, optimizing the success rate of LLM outputs for identifying the correct predefined values for the parameters of interest in each synthetic post. We continued this iterative process until achieving consistent and adequate results (based on gpt-4-0314). Of note, the synthetic sets generated and used for prompt iteration were separate from the set of posts used in our final LLM analyses described in the Results section. This prompt optimization process led to the final version of the prompt used in the subsequent analyses described herein.

### Data Distribution Summary

A summary of our data distribution across sources and their allocation between prompt optimization and validation is provided in Table S1 in Multimedia Appendix 2. We generated >1400 synthetic posts, using small subsets (≤250 posts/iteration) for prompt optimization while reserving 1152 posts for validation. All 370 Twitter/X posts, 290 forum posts, and 956 YouTube posts were used exclusively for validation purposes. Notably, our sampling strategy differed across platforms: while Twitter/X and forum posts were enriched solely for outbreak-related content, the YouTube dataset was deliberately broader and more balanced, combining all 478 conjunctivitis-related YouTube posts containing explicit outbreak or spread-related terms with a random sample of 478 conjunctivitis-related YouTube posts without such terms. This balanced approach for YouTube was designed to enable us to assess LLM performance across a broader spectrum of conjunctivitis-related discussions, including both outbreak-enriched and nonenriched content. All real-world datasets were not used in the prompt optimization phase to ensure an unbiased assessment of LLM performance on genuine social media content. This data distribution strategy allowed us to (1) develop and refine our prompts using a substantial set of synthetic posts; (2) validate LLM performance on a larger, separate set of synthetic posts; and (3) further validate LLM performance on real-world data from multiple social media platforms. By clearly separating our optimization and validation datasets, we aimed to minimize overfitting and provide a robust evaluation of LLM capabilities in extracting epidemiological information from social media posts.

### Optimized LLM Prompt

For our LLM assessment analyses, we used the optimized user prompt for each LLM (with slight variations per LLM to accommodate any LLM-specific technical formatting requirements; Textbox 2). For some APIs that allowed them, we also included a system prompt [59] to convey a public health analyst role to the LLM (Textbox 2). Such a system prompt may reduce the chance that the LLM will respond with helpful advice (such as telling the user to seek medical attention), respond with disclaimers, or refuse to answer the query.

**The prompts used in this study.**

- System prompt: “You are a health analyst for a Department of Public Health. You are summarizing what individuals are saying in social media posts, helping to distinguish reports of rumors, discussions of movies, and so on from reports of actual cases of disease.”
- User prompt: “For every snippet provide the following: how certain are you that this snippet is about a multiperson outbreak of pink eye occurring at the time the snippet was posted? A single case with no other evidence of spread or other infected people should correspond to a somewhat low probability. Suggestions of numerous people affected at one or multiple location or groups impacted (things like \“everyone at...\” or \“the entire district of...\” or \“...something is cancelled\” or \“my work is empty\” or \“something is closed today due to\” or \“school closed\” or \“daycare closed\”) should have a higher probability, and the more people affected, the higher the probability should be. Any obvious conjunctivitis epidemic with more than one person should receive a high score. If it’s about pink eye in non-human animals, then the probability is 0%. If it seems like it is not about a real-life occurrence (for example if it is about dreams, or about fake news, or about rumor, or about a fictional movie or tv shows, or literary fiction, etc.) then assign a probability of zero 0%. Assume all symptoms mentioned are ones that can occur in real life though, even things like \“can’t taste\” or \“can’t smell\” or \“lymph nodes\” are real symptoms. Assume none of these snippets are about fictional characters. Do NOT guess at location, just use the information provided for location. In addition, provide an estimate of the severity on health (mild, moderate, strong, severe, where \“mild\” is not significant, \“moderate\” has some impact on health, \“strong\” has serious health impact and \“severe\” is life-threatening). Also provide the type of outbreak: \“allergic\”, \“infectious\”, \“environmental\” (swimming pools, pollution, toxic spills, smoke, wildfires), or \“infectious-AHC\” (AHC is very severe and typically includes extremely red, bloody or bleeding or blistering eyes, vision loss and other severe symptoms). If there is content about drugs or drug usage (i.e. smoking weed, pot, marijuana, a joint, a bowl, a bong), getting high, sparking up, other slang terms like \“Mary Jane\”, \“bud\”, \“ganga\”, \“reefer\”, \“chronic\”, \“herb\”, \“spliff\”, \“roach\”, \“a j\”) then consider the type of outbreak as \“environmental - drugs\”. If you determine that the cause is infectious, please also tell us whether it is viral or bacterial. Also, tell us the health condition or disease being discussed (e.g. conjunctivitis, flu, broken leg, etc.) In your responses, label each answer. When you tell us the snippet ID, write \“snippet ID:\” followed by the snippet ID. When you tell us the location, write \“location:\” followed by the location, and so forth. Respond in the form of snippet ID, location, a numeric percentage of the probability between 0% and 100%, and then provide a numeric value for the number of people affected based in part on the estimated population of the location and how many of them might be affected (provide a NUMBER of people, do NOT provide a categorical term like low or high, instead provide an actual estimated number), type of outbreak and cause if infectious conjunctivitis, a list of the symptoms, the severity on health, the health condition or disease being discussed, and provide a brief explanation of your answer. Given this single snippet: \“{}\””

### LLM Classifications of Synthetic Posts

After prompt optimization, we generated a final set of 1152 synthetic posts to use in subsequent studies. We submitted the optimized prompt to the selected LLMs and collected their outputs. For the most part, the LLMs responded with semistructured outputs (Textbox 3).

**Example output from a large language model in response to the optimized prompt.**

“**Snippet ID:** K1428, **Location:** household implied, **Probability:** 85%, **Number of People Affected:** 3-5 (assuming a typical number of children in a household), **Type of Outbreak:** Infectious (likely viral), **Symptoms:** Pink eye, **Severity on Health:** Moderate, **Health Condition or Disease:** Conjunctivitis (pink eye), **Explanation:** The snippet describes a household situation where multiple children are sick with pink eye. The phrase ‘filled with sick kids’ suggests that more than one child is affected, indicating a multiperson outbreak within the household. Although the exact number of sick children is not specified, the context implies a significant number. The urgency implied by ‘help!’ indicates concern, but there are no life-threatening symptoms mentioned, so the severity is considered moderate. The cause is likely infectious, probably viral, given the spread among children in a shared living environment.”

After collecting the outputs, applying an LLM chain approach, we used an LLM (refer to Multimedia Appendix 1 for the data extraction prompt) to extract the following variables (among others) into a structured format from the raw, single-paragraph LLM responses: (1) location, (2) probability, (3) the number of people affected, (4) outbreak type, (5) severity, and (6) health conditions. To assess the information provided by the LLMs regarding the health conditions, we used *scispaCy* (The Allen Institute for Artificial Intelligence) for named entity recognition, specifically, the *en_core_sci_lg* model in combination with the Unified Medical Language System vocabulary. This entailed some modifications (eg, the word “croup” needed to be converted to “laryngotracheobronchitis” first to avoid unwanted conversion of “croup” into “group”), and we also used the Python *TextBlob* package to further correct occasional misspellings. It is worth noting that our data extraction methodology involved a 2-step extraction process (initial unstructured LLM responses followed by structured extraction) to balance the need for detailed analysis with structured data requirements. While this approach could potentially introduce minor information loss, it allows for both quantitative analysis and qualitative insights. The use of a specialized named entity recognition tool for health condition extraction leverages domain-specific vocabularies and efficient processing.

### Gold Standard Classifications of Synthetic Posts

The data for this study were obtained from synthetic social media posts, referred to as “synthetic posts,” that were generated to simulate real-world scenarios of outbreak reporting. The gold standard data consisted of epidemic probability scores, severity categorizations, and etiological classifications preassigned to the components of these synthetic posts at the time of their creation. Of the posts assessed by each LLM, most (952/1152, 82.7%) of the posts were about conjunctivitis in one form or another, and a small number (179/1152, 15.5%) were exclusively about other conditions and (19/1152, 1.6%) were about no health conditions).

### Comparing LLM and Gold Standard Classifications

Each LLM’s assessments of the epidemiological characteristics were subsequently validated by comparison to the predefined gold standard values assigned to the synthetic posts. To perform this comparison, we conducted the following statistical analyses.

#### Outbreak Probability

To assess the probability of an outbreak as predicted by the LLM, we calculated the Pearson correlation coefficient between the gold standard probability scores and the model’s assigned probabilities of an outbreak. Synthetic posts labeled as not being about conjunctivitis were excluded to ensure consistency in the analysis. For a more in-depth assessment of performance measures beyond Pearson correlation, we also evaluated the accuracy and calibration of outbreak probability assessments by decomposing errors into bias, calibration error, and resolution [60,61]. Reliability was also measured using regression of observed outcomes on assessed values; a regression slope close to 1 indicates excellent reliability, confirming that assessments closely match observed variations. Bias reflects systematic over- or underassessment, whereas calibration error measures how closely assessments align with actual outcomes across different subgroups (bins). Resolution indicates the ability of assessments to discriminate outcome variations across these groups.

It is important to note that for all additional measures outlined herein, when the outbreak probability preassigned to the synthetic post was <20%, the LLMs often refused to provide further assessments for the prompts beyond the outbreak probability (the LLMs frequently mentioned seeing no reason to continue assessing outbreak characteristics if they did not think that the post was about a true outbreak). Therefore, for the assessment of all relevant variables (ie, except for outbreak probability), we only used the set of posts where the probability score preassigned to the synthetic post was ≥20%. In addition, results from posts that were entirely unrelated to conjunctivitis were only included in our assessments to identify other health conditions mentioned in a post.

#### Outbreak Type

For each of the 4 outbreak types (infectious, allergic, environmentally caused, and AHC-infectious), the sensitivity and specificity of the LLM in identifying the type were compared to the gold standard.

#### Outbreak Size (Number of Cases)

The relationship between the gold standard outbreak size category and the LLMs’ estimated numeric outbreak size was evaluated using the Spearman rank correlation coefficient.

#### Outbreak Severity

We determined the proportion of records in which the gold standard severity was within 1 level of the model’s assigned ordinal severity.

#### Health Conditions

The sensitivity and specificity of the LLM in identifying the following health conditions in each post were compared to the gold standard: conjunctivitis, COVID-19, influenza, gastrointestinal illness, croup, lice infestation, and broken leg.

#### Community Setting

In the context of our study, “community setting” refers to the specific setting or geographic area mentioned in a social media post where an outbreak is reported to occur. This could range from broad locations (eg, “New York City”) to more specific community settings (eg, “Lincoln High School” or “Sunshine Daycare Center”). To assess how accurately LLMs identified and described these community settings, we compared the LLM location outputs to the gold standard predefined location values in our synthetic posts. However, direct string matching was insufficient due to potential variations in describing the same location (eg, “NYC” vs “New York City”). Therefore, we used bidirectional encoder representations from transformers (BERT) to measure the semantic similarity between the LLM-generated location descriptions and the gold standard locations. Specifically, we used mean cosine similarity scoring [62] with BERT. We used the BERT model and tokenizer [63] (accessed via the HuggingFace *transformers* Python library) to tokenize input texts, generate contextual embeddings, and compute cosine similarity between these embeddings. This approach allowed us to capture semantic similarity even when the exact wording differed between community setting text pairs. We calculated the mean cosine similarity across all post pairs to obtain an overall measure of how well the LLMs identified the correct community settings; for example, if the gold standard location was “Springfield Elementary School,” and the LLM output was “local primary school in Springfield,” BERT would likely yield a high similarity score despite the lack of exact string matching. This method allowed us to quantitatively assess the LLMs’ ability to correctly interpret and report the community settings mentioned in the posts, accounting for variations in language and specificity.

### Cross-Model Validations: Synthetic Posts

LLMs are increasingly being used in all fields, including health [51-53,64]. A key challenge in using LLMs for such specialized tasks, including for epidemic surveillance, is the need to validate their outputs across many different examples within the domain. This becomes more significant if they start being used for ongoing surveillance. While human expert validation is the gold standard, it is impractical to have experts review large volumes of LLM classifications. To overcome this key challenge, numerous solutions providing a scalable validation approach have been proposed to both improve LLM assessments (using ensemble or chained LLMs) and to compare outputs across multiple independent LLMs [45,51,52,65-70]. This builds on the principle that agreement between independently developed LLMs increases confidence in the results, while disagreement flags potential issues requiring human review. In this manner, there is potential to complement human validation efforts in an affordable and statistically centered approach.

In our study, we implemented this validation approach by comparing GPT-4’s classifications of synthetic posts against those from other leading LLM platforms. Specifically, after identifying GPT-4 (gpt-4-0314; referred to as GPT-4 hereinafter) as one of the more successful LLMs in classifying epidemiological characteristics from synthetic posts, we validated its performance by comparing its classifications to those from other independent LLMs using the same set of 1152 synthetic posts as described in the preceding subsection (Comparing LLM and Gold Standard Classifications). This allowed us to determine whether different LLMs, developed by separate companies using different training approaches, would reach similar conclusions when analyzing the same content. The methods used and components assessed (outbreak probability, outbreak type, outbreak size, outbreak severity, and other health conditions) were identical to those described previously, with the exception that the standard values in these assessments were the GPT-4 extracted values, rather than the gold standard predefined values of the synthetic posts. The ability of different leading LLMs to successfully classify epidemiological characteristics similarly to GPT-4 would suggest that we could also apply this approach to validate the results of LLM classifications of the epidemiological characteristics of real-world posts by comparing the results from GPT-4 to those from other unrelated LLMs. This consideration led to our next comparative assessment, described in the following subsection.

### Cross-Model Validations: Real-World Posts

We assessed the ability of LLMs to classify epidemiological characteristics from a set of real-world Twitter/X posts, a set of forum posts, and a set of YouTube posts using GPT-4 as a surrogate standard because there was no gold standard for the classification of parameters in these posts. The methods used and components assessed (outbreak probability, outbreak type, outbreak size, outbreak severity, and other health conditions) were identical to those described previously, with the exception that the standard values for these assessments were the GPT-4–extracted values for the Twitter/X, forum, and YouTube posts. The results from other LLMs were compared to these GPT-4 values. Similar results across LLMs from different companies and platforms could help validate the GPT-4 results, but an additional approach would be to validate the GPT-4 results using human raters. This approach is described in the next subsection.

### Human Validation of LLM Classifications

#### Overview

Having established the success of GPT-4 in extracting epidemiological characteristics, validated by other LLMs, from real-world posts, we then conducted validation studies using human raters. These raters classified epidemiological characteristics of real-world posts using an approach similar to that of our prior study [18] but with a larger number of participants and more variables to be rated. We selected 2 groups of human evaluators to provide the gold standard assessments of the LLM results. The two groups consisted of (1) nonexpert raters (2 individuals with no medical training but with extensive experience of using and studying social media) and (2) expert raters (2 board-certified ophthalmologists with at least 5 years of clinical experience in ocular surface diseases [expert ophthalmologists]). This dual-perspective approach, incorporating both nonexpert raters and ophthalmologist raters, was to ensure that some raters brought strong familiarity with social media communication patterns, language, and context, including an understanding of current slang, hashtag use, and typical ways in which health concerns might be expressed on these platforms, while the ophthalmologists ensured that some raters had high-quality clinical expertise.

#### Human Rater Assessment Sessions

Before classifying posts, each human rater participated in calibration and alignment sessions conducted via Zoom (Zoom Video Communications, Inc) with a study team member, including the use of Qualtrics surveys (Qualtrics International Inc) for the classification tasks. This was to familiarize the raters with the classification tasks conducted using Qualtrics surveys, ensure alignment and consistency in their assessments, and introduce them to the nuances of social media language (refer to Multimedia Appendix 3 for more details on these sessions). After the calibration sessions, for each epidemiological characteristic, the raters were again provided the original LLM prompts and the human instructions for each section. They then independently assessed a set of test posts in a separate Qualtrics survey (excluding any posts used in the calibrations sessions). The raters assigned classifications and probability scores without knowledge of other raters’ or GPT-4’s classifications, ensuring unbiased assessments.

#### Human Rater Classifications of Posts Using Qualtrics

The nonexpert human raters classified posts across 5 characteristics: outbreak probability, outbreak type, outbreak size, outbreak severity, and other health conditions mentioned. The expert raters—practicing ophthalmologists trained in ocular surface disease—classified the same set of test posts, focusing on outbreak type and outbreak severity as well as 1 additional characteristic: etiology. All raters used the same questions and response options. Details are presented in Textbox 4, and the Qualtrics instructions for each item are presented in Multimedia Appendix 3.

**Summary of the characteristics assessed and the associated questions and response options used by human raters in Qualtrics.**

- Outbreak probability: for 72 posts (synthetic posts: n=24, 33%; Twitter/X posts: n=24, 33%; and forum posts: n=24, 33%), raters were asked, “How certain are you that this snippet is about a multiperson outbreak of pink eye occurring at the time the snippet was posted?” and provided with response options ranging from 0% to 100% in increments of 10%.
- Outbreak type: for 90 posts (synthetic posts: n=38, 42%; Twitter/X posts: n=26, 29%; and forum posts: n=26, 29%), raters selected from the following options: “NOT SPECIFIED,” “ALLERGIC,” “INFECTIOUS,” “ENVIRONMENTAL,” and “AHC-INFECTIOUS.”
- Outbreak size: the same 90 posts were evaluated by asking raters to provide their best numerical estimate of the individuals affected based on the post content, with the option to input “N” if the size was not defined.
- Outbreak severity: for the same 90 posts, raters chose one of the following response options: “NOT SPECIFIED,” “MILD,” “MODERATE,” “STRONG,” and “SEVERE.”
- Health conditions mentioned: for 50 (4.34%) of the 1152 synthetic posts, raters were asked to list any health conditions discussed in the post, excluding individual symptoms unless considered a known health condition.
- Etiology: for the same 90 posts used for the outbreak type assessment, we also used a modified prompt with etiology-specific language to elicit responses from GPT-4. In Qualtrics, the 2 expert raters—practicing ophthalmologists trained in ocular surface disease—were given instructions to assign an infectious or noninfectious cause for each of the 90 posts, choosing from the same 6 etiology categories as provided in the prompt (“NOT INFECTIOUS,” “INFECTIOUS: UNSPECIFIED,” “BACTERIAL,” “VIRAL: UNSPECIFIED,” “VIRAL: COVID-19,” “VIRAL: AHC”). The modified GPT-4 etiology prompt is provided in
  Multimedia Appendix 3
  .

#### Correlation, Sensitivity, and Specificity Analyses of Human Rater Validation Results

The assessment of the human rater classifications of these components (outbreak probability, outbreak type, outbreak size, outbreak severity, and other health conditions) followed the same procedures described previously, with the exception that the human rater values were compared to those generated by GPT-4. For etiology, the classifications provided by the expert ophthalmologists were treated as the gold standard. Sensitivity was defined as the model’s ability to correctly identify positive cases and specificity as its ability to correctly identify negative cases. The *bincmp* function was used to calculate sensitivity and specificity for each etiological factor. This function compared the binary assessments generated by GPT-4 against the ophthalmologists’ assessments. For any result in which the denominator was <5, we present results as a ratio rather than as a fraction. For etiology, outputs were calculated both across the combined set of posts and separately by each source type: synthetic posts, Twitter/X posts, and forum posts.

#### Interrater Reliability Analysis of Human Rater Validation Results

To assess interrater reliability between human raters and LLMs, we calculated intraclass correlation coefficients (ICCs) for continuous measures (outbreak probability, size, and severity) and Fleiss κ for categorical classifications (outbreak type and other health conditions). For analysis of size, because of the wide range of possible values, we used the pseudologarithmic transformation arcsinh(x/2), which is asymptotically logarithmic but defined at 0. ICCs were calculated separately for (1) agreement between nonexpert raters, (2) agreement between expert ophthalmologists, (3) agreement between human raters and LLM assessments, and (4) overall agreement across all raters. For categorical classifications, we used Fleiss κ to assess agreement between multiple raters, calculating values for all raters combined and for subgroups (experts only and nonexperts only). The results of these analyses are presented in Table S1 in Multimedia Appendix 4.

### Additional Methods Considerations

#### CI Calculations

To calculate the 95% CIs for all sensitivity, specificity, and percentage agreement results in our study, the exact Clopper-Pearson method was used. To calculate the 95% CIs for all correlation coefficients shown, the Fisher *z* transformation method was used.

#### Software and Environment

All statistical analyses were conducted using R (version 4.2.0 for Macintosh). The *scispaCy* package and all API calls were conducted through Python (version 3.0; Python Software Foundation) on an Ubuntu Linux-based cloud server.

### Ethical Considerations

This study was approved by the University of California San Francisco Institutional Review Board (14-14743). All social media data used in this study were collected from publicly available sources through the Brandwatch platform. To ensure privacy and confidentiality, only the textual content of Twitter/X, forum, and YouTube was analyzed; associated images, videos, and user profile information were excluded. Where direct quotes from real-world social media posts are included in the manuscript, we have redacted identifiers. The synthetic posts created for this study do not contain any real user data.

## Results

### Summary of LLM Responses and Performance

The overall ability of the LLMs to respond in the format we desired was high because all 7 LLMs yielded usable responses for all 1152 synthetic posts, all 370 Twitter/X posts, and all 290 forum posts, with a single exception: GPT-4o failed to produce a usable result for 1 forum post. As in our previous study using GPT-4 with Twitter/X posts [18], in this study, we observed that the ability of multiple LLMs to summarize the content and characterize epidemiological characteristics across the synthetic, Twitter/X, and forum posts seemed generally reliable and surprisingly nuanced, although performance varied by model. Table 1 presents a selection of GPT-4’s prompt responses for synthetic, Twitter/X, and forum posts. To ensure privacy, identifiers in posts have been redacted.

**Table 1:** Selected examples of GPT-4 prompt responses for synthetic, Twitter/X, and forum posts. Identifying details in Twitter/X and forum posts have been redacted (indicated with square brackets). In some cases, longer explanatory content has been truncated (indicated with ellipses) to save space. Posts were selected to illustrate a range of epidemiological characteristics across the 3 post sources.

Sources and original snippets	GPT-4 classifications	GPT-4 explanation
Source: synthetic post Snippet: “our whole household attedning Montepuez, Mozambique complinaing about diarrhea with headache and pain in limbs combined with emergency room”	Probability: 0% Type: not applicable Size: 0 Severity: not applicable Etiology: not applicable	The snippet does not mention pink eye or conjunctivitis, so the probability of a multiperson outbreak of pink eye is 0%. The symptoms mentioned are not related to pink eye. The health condition being discussed is not specified, but the symptoms suggest a gastrointestinal issue. The severity on health is not specified in the snippet.
Source: synthetic post Snippet: “in my dream in Epecuen, Argentina complinaing about pinkneye with hayfever and itchy and swollen”	Probability: 0% Type: not applicable Size: 0 Severity: not applicable Etiology: not applicable	The snippet mentions a dream, which indicates that this is not a real-life occurrence. Therefore, the probability of a multiperson outbreak is 0%. No real people are affected, and no real symptoms or health conditions are being discussed.
Source: synthetic post Snippet: “lotta peeps attedning The Yellowstone National Park seemt to have conjunctivitis with allergy and Intense itching with concerned”	Probability: 70% Type: allergic Size: hundreds Severity: mild Etiology: not applicable	The snippet suggests that multiple people visiting Yellowstone National Park are experiencing symptoms of conjunctivitis, specifically intense itching, which is often associated with an allergic reaction. However, without more specific information, it’s difficult to determine the exact number of people affected or the cause of the outbreak. The severity is considered mild as conjunctivitis, while uncomfortable, is typically not life-threatening.
Source: synthetic post Snippet: “vision problems with clearly an outbreak in Bourbon Street complinaing about pink eyes with bleeding and fluid-filled, blister-like growths on top of the eye surface (chemosis) with eyelid swelling and pain and decreased vision”	Probability: 90% Type: infectious-AHC Size: 100 Severity: severe Etiology: virus	The snippet describes an outbreak of pink eye with severe symptoms such as bleeding, blister-like growths, and decreased vision, which are indicative of Acute Hemorrhagic Conjunctivitis (AHC), a severe form of conjunctivitis. The location is specified as Bourbon Street, and the use of the term “outbreak” suggests that multiple people are affected. Therefore...
Source: forum post Snippet: “I just called my ped about the same thing! He said that viral pink eye is spreading around right now. My [child’s name] has had it for a week.”	Probability: 70% Type: infectious Size: 2 Severity: mild Etiology: viral	The snippet suggests that there is a current outbreak of viral pink eye, as confirmed by a pediatrician. The user’s child has been affected, and it’s implied that others in the community may also be affected. However, without more specific information about the location or the number of people affected, it’s difficult to provide a more accurate estimate. Pink eye is typically a mild condition, although it can be uncomfortable and highly contagious.
Source: forum post Snippet: “[T]o the rule, but they told us blocked tear duct and it was bacterial conjunctivitis. She ended up spreading it to big sister and me!”	Probability: 100% Type: infectious Size: 3 Severity: moderate Etiology: bacterial	The snippet clearly mentions that the person had bacterial conjunctivitis and it was spread to two other people. This indicates a small-scale outbreak of an infectious disease. The severity is considered moderate as bacterial conjunctivitis can cause discomfort and temporary vision problems but is not typically life-threatening.
Source: forum post Snippet: “[K]now Pink eye that easily infectious. Hopefully everyone recovers with no lasting damage. I just saw that India had an outbreak in August too, it sounds like it spread to Pakistan. Damn, I saw it spread to Vietnam too Conjunctivitis (pink eye) sees a spike in cases in Vietnam, India and Pakistan Oh my God. Close all travels. And I do mean ALL. ALL...”	Probability: 90% Type: infectious Size: thousands Severity: moderate Etiology: not specified	The snippet mentions an outbreak of pink eye in multiple countries, suggesting a widespread infectious event. However, without more specific information, it’s difficult to determine the exact number of people affected or the cause of the outbreak.
Source: Twitter/X post Snippet: “[@username] god next thing we know there's there’s gonna be someone spreading pink eye around...”	Probability: 10% Type: infectious Size: 1 Severity: not specified Etiology: not specified	The snippet suggests a hypothetical situation of someone spreading pink eye, but it does not provide concrete evidence of an actual outbreak. The “\/j\” at the end of the post indicates that the user is joking.
Source: Twitter/X post Snippet: “When it rains it pours 😭😭 Pink eye-3 [surname family-1]. Here’s hoping it’s done spreading and we can finally kick its butt!’”	Probability: 70% Type: infectious Size: 4 Severity: moderate Etiology: not specified	The snippet suggests a family (the [surname] family) has been affected by pink eye, with at least three members infected. The phrase “Here’s hoping it’s done spreading” suggests it’s an infectious outbreak. However...
Source: Twitter/X post Snippet: “There’s some weird virus going around that has all the cold/flu symptoms, fever, super sore throat, ear pain and pink eye. It has ran through my household and I’m feeling like death right now. 😭😭😭”	Probability: 80% Type: infectious Size: 5 Severity: strong Etiology: viral	The snippet suggests a multi-person outbreak of pink eye, likely viral given the accompanying symptoms of cold/flu, fever, sore throat, and ear pain. The user mentions it has ran through my household

### LLM Validation Summary

#### Overview

**Figure 1:**
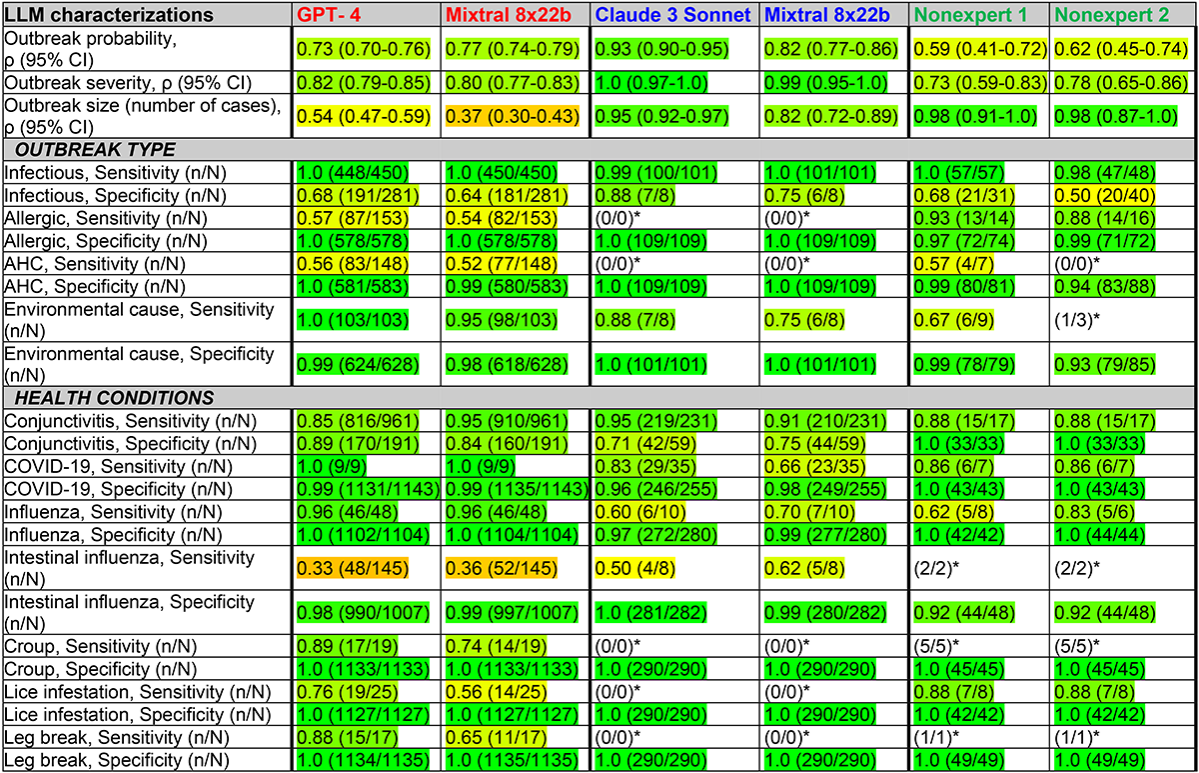
Characterizing epidemics. On the basis of the content of synthetic and real-world social media posts (forums and Twitter/X), large language models (LLMs) can characterize outbreak probability, type, size, severity, and other health conditions. We tested 7 LLMs; the high-performing LLM results are shown in columns 2 to 5. Rows 1 to 3 present Spearman correlations; from row 4 onward, sensitivity and specificity are shown. Cell colors indicate values ranging from 0.0 (red) to 0.5 (yellow) to 1.0 (green). Columns 2 and 3 present the results of LLM validation against the gold standard. With known preclassified values of epidemiological characteristics (severity, etc) in synthetic posts, the LLMs’ abilities to classify these characteristics from these posts’ content were assessed. Columns 4 and 5 present the results of comparative intermodel validations. In the absence of predefined known values of synthetic posts, we used an LLM (GPT-4) to help validate the results from other LLMs’ characterizations of epidemics from synthetic and real-world posts (the results shown are for the classifications of forum posts; a comparison of results from other LLMs to those from GPT-4 is shown). Columns 6 and 7 present the results of human rater validations. In the absence of predefined known values, human raters can assign values that can be compared to an LLM’s characterization of epidemics from posts. Comparisons between GPT-4 and the classifications provided by 2 nonexpert human raters are shown for synthetic posts as well as for real-world Twitter/X and Forum Posts. AHC: acute hemorrhagic conjunctivitis. *Results with a denominator of ≤5 are shown as fractions.

The results for the top-performing LLMs for many of the different comparisons are shown in Figure 1, highlighting their ability to accurately classify key characteristics of potential epidemics from social media content, specifically outbreak probability, type, size, severity, and other health conditions. Figure 1 includes 3 sets of validation comparisons: (1) LLMs classifications against known gold-standard values of synthetic posts, (2) LLMs classifications of real world posts compared to one another, and (3) humans' compared to LLMs' classifications of real world posts. For each comparison, results are shown for the 2 top-performing LLMs. Complete results for all LLMs across each comparison are presented in Multimedia Appendix 4.

#### Validating LLMs Against Gold Classifications

We first assessed the ability of the LLMs to extract outbreak characteristics from synthetic posts where the ground truth was known. We found that when evaluated against synthetic data, the LLMs demonstrated strong performance in predicting outbreak probability, size, and severity, achieving high correlations with predefined values (Figure 1; columns 2 and 3); for example, GPT-4 and Mixtral 8x22b achieved high correlations of 0.73 and 0.77, respectively, with the true outbreak probability, indicating a strong ability to distinguish between likely and unlikely outbreaks. Similarly, both excelled in estimating outbreak severity, achieving correlations of 0.82 and 0.80, respectively. Although still correlated, their performance on size estimation was notably lower than their performance on outbreak likelihood and severity. Results for all 7 LLMs are shown in Table S2 in Multimedia Appendix 4. We also assessed the LLMs’ ability to determine community setting (results not shown in the table) and found that they were fairly successful; for example, comparing the “location” values extracted by LlaMa 3 70B (using mean cosine similarity scoring with BERT) to the predefined community settings, we found that community setting was ascertained well overall across all combined location sizes (country, state, house, etc; mean 0.91, SD 0.16) and for more specific community settings as well, for example, daycare centers (mean 0.88, SD 0.16), colleges and dormitories (mean 0.92, SD 0.13), and homes and condominiums (mean 0.77, SD 0.22).

In our more in-depth assessment of the performance measures of bias, calibration error, resolution, and reliability regarding our key measure of outbreak probability, GPT-4 and Claude 3 Sonnet demonstrated the most effective performance, although significant room for improvement remains. GPT-4 showed the lowest bias (8.8 percentage points) and calibration error (16.6 percentage points), indicating relatively better alignment with actual outcomes compared to other models. Claude 3 Sonnet performed similarly, with a bias of 11.5 percentage points and a calibration error of 17.7 percentage points. Both models exhibited moderate resolution (approximately 25 percentage points), showing some ability to distinguish between different outbreak scenarios. Their reliability coefficients (Claude 3 Sonnet at 0.66 and GPT-4 at 0.64), although better than those of others, still suggest only moderate consistency between assessed probabilities and actual outcomes. Overall, while GPT-4 and Claude 3 Sonnet were the strongest performers in this analysis, their performance should be regarded as modest, highlighting the potential for further calibration and refinement in real-world applications. Bias, calibration error, resolution, and reliability performance measure results for the 7 LLMs included in this study are shown in Table S3 in Multimedia Appendix 4.

#### Cross-Model Validations Using Multisource Real Posts

Moving beyond synthetic data, we validated LLM performance on real-world forum posts by comparing each model’s outputs to those of GPT-4. We found that the LLMs demonstrated high correlation with GPT-4’s assessments across various outbreak characteristics (Figure 1, columns 4 and 5 in Multimedia Appendix 4); for instance, both Claude 3 Sonnet and Mixtral 8x22b showed high correlations with GPT-4 in assessing outbreak likelihood, with correlation coefficients of 0.93 and 0.82, respectively. When evaluating the models on Twitter/X data, the correlations were slightly lower, with Claude 3 Sonnet at 0.82 and Mixtral 8x22b at 0.74 for outbreak likelihood. Analysis of the YouTube dataset showed that the LLMs maintained strong performance in classifying outbreak-related content, with Mixtral 8x22b showing high correlation with GPT-4’s outbreak probability assessments (0.75). However, outbreak size estimates from YouTube posts showed lower correlation for Mixtral 8x22b (0.54) compared to Twitter/X (0.84) and forum posts (0.82). For noninfectious classification by Mixtral 8x22b, the identification of outbreak types maintained high specificity across categories for YouTube posts but showed worse sensitivity than for Twitter/X and forum posts. For detailed results of all three social media platforms, see Table S4, S5, and S6, respectively of Multimedia Appendix 4.

#### Human Rater Validations of LLM Classifications

To understand whether the insights identified by LLMs could be consistently recognized by humans familiar with social media but not clinical experts, nonspecialist human raters assessed the same social media posts evaluated by GPT-4. Across both synthetic and real-world data, the raters showed substantial agreement with GPT-4 but with some discrepancies (Figure 1; columns 6 and 7). For outbreak type, the raters achieved a mean sensitivity of 0.80 (SE 0.055) and mean specificity of 0.84 (SE 0.020), indicating strong agreement with GPT-4 in categorizing outbreaks as infectious, allergic, or environmentally caused. However, there were notable exceptions, such as in identifying AHC from real-world posts, where all 4 human rater's sensitivity was considerably lower and varied. Our ICC and κ analyses, described in the next subsection, further quantified these patterns of agreement.

#### Interrater Reliability Analysis

As shown in Table S1 in Multimedia Appendix 4, comprehensive interrater reliability analysis revealed varying levels of agreement across different assessment types and rater groups. For outbreak probability, the nonexpert raters showed good internal consistency (ICC=0.76, 95% CI 0.65-0.84), while agreement between the nonexperts and the LLM was moderate (ICC=0.63, 95% CI 0.47-0.75). For outbreak severity, agreement between the LLM and the clinical experts was substantial (ICC=0.69, 95% CI 0.56-0.79), notably higher than that between the nonexpert raters (ICC=0.18, 95% CI −0.08 to 0.50) or between the clinical experts (ICC=0.38, 95% CI 0.05-0.61). Agreement between the clinical experts and nonexperts was moderate (ICC=0.52, 95% CI 0.30-0.68), while LLM agreement with all clinical assessments combined showed strong reliability (ICC=0.70, 95% CI 0.58-0.79).

For categorical classifications, agreement varied by condition type. Allergic conjunctivitis classification showed strong agreement among all raters (κ=0.78, 95% CI 0.67-0.88), with nonexperts showing particularly high consistency (κ=0.84, 95% CI 0.66-0.97). By contrast, AHC classification showed poor agreement across all groups (κ=0.37, 95% CI 0.24-0.45). For infectious cases, the nonexperts demonstrated moderate agreement (κ=0.58, 95% CI 0.39-0.74), while clinical experts showed fair agreement (κ=0.25, 95% CI 0.01-0.46). The classification of other health conditions showed excellent agreement, with perfect agreement for some conditions (croup and broken leg: κ=1.0) and near-perfect agreement for others (lice infestation: κ=0.95, 95% CI 0.81-1.0). While a detailed analysis of the human-AI discrepancies was beyond the scope of this initial validation study, these findings highlight an important area for future research: understanding how human expertise and AI capabilities can be optimally combined for public health surveillance. The observed variations between human experts and GPT-4 suggest that human oversight of GPT-4 classifications of AHC could help reduce overcalling of high-risk outbreaks while maintaining the efficiency benefits of automated screening.

### Expert Validation of Etiology

**Figure 2:**
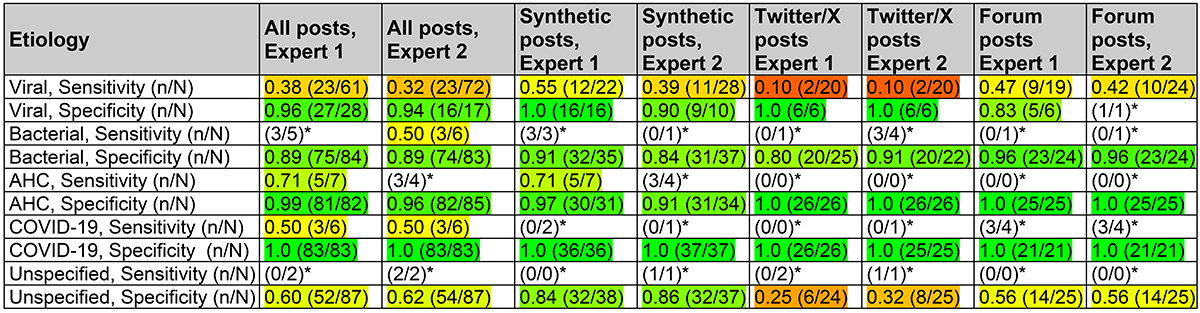
Specialist expert validations of conjunctivitis etiology. We used the same data and method as presented in Figure 1 (columns 6 and 7), but we compared ophthalmologist expert–assigned conjunctivitis etiology and GPT-4–assigned etiology. For the set of 90 posts (synthetic posts: n=38, 42%; Twitter/X posts: n=26, 29%; and forum posts: n=26, 29%; Figure 1), etiology outputs from a modified large language model (GPT-4) prompt were compared to those of the 2 ophthalmologists, and when comparing each to GPT-4, the ophthalmologist was treated as the gold standard. All rows report sensitivity and specificity. Cell colors indicate values ranging from 0.0 (red) to 0.5 (yellow) to 1.0 (green). Results were calculated for all 3 sources of posts combined (columns 1 and 2), as well as for each individual source (synthetic posts: columns 3 and 4; Twitter/X posts: columns 5 and 6; and forum posts: columns 7 and 8). AHC: acute hemorrhagic conjunctivitis. *Results with a denominator of ≤5 are shown as fractions.

We further evaluated the ability of GPT-4 to arrive at the same conclusions as the 2 expert ophthalmologists regarding the cause of infectious conjunctivitis from social media posts. Across all etiology types, GPT-4 showed high agreement with the experts’ assessments (Figure 2), particularly in ruling out conditions (high specificity). For viral conjunctivitis, GPT-4 mirrored the experts’ high specificity with a value of 0.96; however, GPT-4’s sensitivity for identifying cases where the experts did identify viral conjunctivitis was lower at 0.38. This suggests that while GPT-4 is highly reliable in excluding viral conjunctivitis when it is not present, it might miss some actual cases. GPT-4 had very high agreement with an expert’s identification of AHC, a rare form of conjunctivitis with high severity, achieving a specificity of 0.99 and a sensitivity of 0.71. The performance varied across different data sources: for combined data, GPT-4 had a mean sensitivity of 0.53 (SE 0.16) and mean specificity of 0.89 (SE 0.036). This performance declined slightly for Twitter/X posts, with sensitivity dropping to 0.10 (SE 0.089) and specificity to 0.81 (SE 0.052), suggesting that the limited context in these posts makes it harder for GPT-4 to identify cases. Conversely, GPT-4 performed better on synthetic data (sensitivity=0.63, SE 0.10 and specificity=0.94, SE 0.05), likely because the synthetic data were more standardized and potentially richer in relevant clinical information. Notably, GPT-4’s performance on forum posts (sensitivity=0.47, SE 0.14 and specificity=0.87, SE 0.068) fell between its performance on Twitter/X and synthetic posts.

### Impact of Prompt Optimization on LLM Performance

In this study, we required 3 main rounds of prompt optimization before arriving at the final prompt used in our analyses. After completing the primary analyses, we conducted an experiment to assess whether the performance improved with the final prompt compared to the earlier iterations. Specifically, we ran the final set of 1152 synthetic posts through each of the 3 preliminary versions of the prompt as well as the final prompt. We then compared a key outcome: outbreak likelihood. The correlation coefficients (with 95% CIs) for GPT-4 classifications of the posts compared to the gold standard values across prompt iterations (based on gpt-4-0413) were as follows (the preliminary versions of the prompt showed slightly lower correlations):

- Prompt 1: 0.71, 95% CI 0.67-0.74
- Prompt 2: 0.75, 95% CI 0.73-0.78
- Prompt 3: 0.75, 95% CI 0.72-0.78
- Final prompt: 0.76, 95% CI 0.73-0.79

We conducted 2 additional assessments. First, we provided only the portion of the final prompt that was specific to outbreak likelihood. For GPT-4, the outcome using this more focused individual question (0.72, 95% CI 0.68-0.75) was not better than the outcome when using the full comprehensive prompt (0.76, 95% CI 0.73-0.79). Second, we inverted the order of the prompt, providing the post content at the beginning of the prompt rather than at the end. For GPT-4, this inversion resulted in a notably lower correlation (0.63, 95% CI 0.60-0.67) compared to the original structure (0.76, 95% CI 0.73-0.79).

## Discussion

### Principal Findings

The main findings of this study demonstrate that top-performing LLMs can extract and classify key epidemiological characteristics from social media post content about conjunctivitis outbreaks. Specifically, we found that LLMs such as GPT-4 and Mixtral 8x22b showed strong performance in predicting outbreak probability, size, and severity from synthetic and real-world social media data. There was high agreement between different LLMs in classifying outbreak characteristics, supporting the reliability of these models for public health monitoring. Human validation generally agreed with LLM classifications, although there were some notable discrepancies in identifying rare or severe conditions. Specialist validation revealed high specificity but a range of sensitivities in identifying different types of conjunctivitis etiology from social media posts. These findings suggest that LLMs have significant potential as tools for early outbreak detection and characterization in public health surveillance, while also underscoring the importance of combining LLM capabilities with human expertise.

In this study, our aim was to achieve several key objectives related to the use of LLMs in the field of infoveillance. Specifically, we sought to determine whether an LLM could accurately classify significant epidemiological features from individual social media posts, including data from other platforms such as web-based forums. The epidemiological characteristics of interest included the probability of an outbreak; the number of people affected; the presence of other health conditions; and the characteristics of conjunctivitis cases such as type, severity, and etiology. Our general hypothesis was that the correlation, sensitivity, and specificity results would vary among different LLMs. We anticipated that some LLMs would outperform others and that certain simpler features (eg, outbreak probability or type) would be classified more accurately than more complex features (eg, severity or etiology).

Overall, several key findings largely supported our hypotheses: we found that several LLMs from different developers performed quite well in identifying many of the characteristics being assessed, demonstrating their capability to discern numerous epidemiological characteristics of outbreaks from both synthetic and real social media posts, including forum posts, which we had not studied before [18]. This includes validation against known values derived from synthetic datasets or through human expert assessments. The top-performing LLMs were able to extract approximate outbreak probability, size, severity, and, to a lesser extent, outbreak type and potentially etiology from social media posts in many cases.

### Other Findings

#### Validating LLMs Against Known Values of Synthetic Posts

##### Overview

When we evaluated the ability of LLMs to accurately classify outbreak characteristics based on synthetic posts with known epidemiological features, several performed well; for example, GPT-4 showed a strong ability, with a correlation of 0.73, indicating its effectiveness in distinguishing between potential and nonpotential outbreaks; and Mixtral 8x22b excelled in estimating outbreak size, achieving a correlation of 0.82. The results underscore their potential utility in early detection scenarios where rapid assessment is crucial. This validation against known values underscores LLMs’ potential as reliable tools in public health monitoring, offering a scalable solution for early outbreak detection and assessment without the need for extensive manual data analysis.

##### Limitations

The primary limitation of this portion of our study is the reliance on synthetic data, which might not always capture the complexity and variability of real-world data. The models’ performance might differ when exposed to less controlled, more diverse datasets. Future research could focus on enhancing LLMs’ training with diverse and complex synthetic datasets that mirror real-world variability in outbreak reports. As we have noted, another limitation of our approach, in the absence of sufficient gold standard conjunctivitis epidemics is that we have not shown that our assessed outbreak probabilities (which may be interpreted as being similar to Bayesian degrees of belief) are calibrated. In other words, we cannot conclude that among Twitter/X posts with assessed outbreak probability x, the relative frequency of true outbreak probability is x. Regarding prompt optimization, while our current prompt format has proven effective in our research context, future studies should specifically address prompt engineering techniques, including experiments with more concise and structured prompts, to assess for further optimized LLM performance.

#### Comparative Validations Using Real Posts From Multiple Sources

##### Overview

We evaluated and compared the ability of LLMs to classify epidemiological characteristics from real-world data, comparing the performance of LLMs such as Claude 3 Sonnet and Mixtral 8x22b against GPT-4 and found that LLMs developed by independent vendors showed high agreement in assessing components such as outbreak likelihood, with Claude 3 Sonnet and Mixtral 8x22b achieving correlations of 0.93 and 0.82, respectively, when evaluating forum posts. The high degree of consistency across different LLMs when analyzing real-world data underscores the reliability of using these models as supplementary tools for outbreak detection and characterization. This method of multimodel validation not only bolsters the confidence in the findings of an LLM but also suggests a potentially streamlined, automated approach to cross-validate epidemiological results from LLM assessments, which traditionally relies heavily on human expertise and intervention.

##### Limitations

Variations in performance across different social media sources such as Twitter/X and forums indicate that LLMs might be sensitive to the format and quality of the input data, which can limit their generalizability. The YouTube analysis highlighted platform-specific challenges in extracting epidemiological information from social media. While our sampling strategy successfully balanced outbreak-related and non–outbreak-related YouTube content, the informal nature of the video comments may have affected the LLMs’ ability to accurately assess certain characteristics, particularly outbreak size, compared to the Twitter/X and forum content. Future research should examine how different social media formats influence the extraction of epidemiological information. Studies should also explore the development of platform-agnostic LLMs that maintain high accuracy across various social media platforms, enhancing their utility in diverse public health contexts. In addition, individual LLMs may exhibit inherent bias. To further improve our models and potentially help mitigate any such bias, in future studies of a larger number of LLMs, we could take advantage of the unique strengths and weaknesses of each LLM to generate an ensemble model and assess its overall ability to improve both sensitivity and specificity. In this study, we also did not include any open-weight LLMs, which offer advantages such as flexibility of local deployment, independence from vendors, cost control, and potential for fine-tuning. Including them in a future ensemble model study (of LLMs spanning a wide range of costs) could help investigate trade-offs between performance and cost.

#### Human Rater Validations of LLM Classifications

##### Overview

In our human validation studies, we found substantial consistency in some areas and significant discrepancies in others; for instance, while the human raters generally agreed with GPT-4 in categorizing outbreaks as infectious, allergic, or environmentally caused, there were notable inconsistencies in the sensitivity and specificity values, especially for more severe or rare conditions. Such human rater inconsistencies align with established clinical literature; for example, a systematic review by Azari and Barney [71] demonstrated that even in clinical settings with direct patient examination, distinguishing between types of conjunctivitis, such as infectious versus allergic, is inherently challenging and often requires laboratory confirmation [72]. However, human raters and GPT-4 showed a high level of agreement in identifying infectious outbreaks, with sensitivity values close to 1.0 across different LLM and human assessments. This consistency suggests that both humans and LLMs are effective at recognizing clear signs of infectious diseases in social media posts. This consistency supports the use of LLMs as a tool to assist public health monitoring and potentially lighten the load on human analysts. Overall, our dual validation approach using both clinical experts and social media specialists proved valuable because both groups showed strong agreement with LLM classifications while bringing complementary expertise in reconciling medical assessment validations with social media communication patterns.

In our assessment of interrater reliability, the ICC and κ analyses (Table S1 in Multimedia Appendix 4) revealed important patterns in agreement between different types of raters. Nonexpert raters showed stronger agreement with each other than with LLMs for outbreak probability assessment, while clinical experts showed better agreement with LLMs for severity assessment. This suggests that LLMs may be capturing some aspects of clinical judgment that align more closely with expert assessment than with layperson interpretation. Notably, while both expert and nonexpert human raters showed only fair internal agreement on severity (ICC=0.38 and ICC=0.18, respectively), the LLMs achieved moderate to substantial agreement with both groups (ICC=0.69 with experts and ICC=0.59 with nonexperts). We found poor agreement on AHC classification (κ=0.37) across all raters, but there were few cases assessed as AHC by the raters.

Comparing our correlation coefficients and interrater reliability measures (ICC and κ) provides complementary insights into LLM performance. While correlations (Table S7 in Multimedia Appendix 4) measured the strength and direction of relationships between classifications, showing, for example, strong correlations between GPT-4 and human assessments for outbreak size (0.98-1.0), the ICC and κ analyses (Table S1 in Multimedia Appendix 4) specifically quantified agreement levels between raters. This distinction proved particularly informative for severity assessments, where moderate correlations between the LLM and nonexperts (0.73-0.78) and between the LLM and experts (0.98-0.99) suggested general alignment in ranking severity (Table S7 in Multimedia Appendix 4); however, lower ICC values (0.18 between nonexperts and 0.38 between experts) revealed challenges in achieving exact agreement on severity levels among human raters (Table S1 in Multimedia Appendix 4). Similarly, while sensitivity and specificity metrics demonstrated GPT-4’s ability to correctly identify specific conditions (eg, sensitivity=0.88-1.0 for conjunctivitis), κ values provided insight into the overall reliability of these classifications across multiple raters (κ=0.94 for conjunctivitis). These complementary statistical approaches revealed that while LLMs can effectively rank and identify conditions (high correlations as well as sensitivity and specificity), achieving consistent absolute classifications even across different human rater types remains challenging for some characteristics (lower ICC and κ values), particularly for more nuanced assessments of social media content, such as AHC identification.

##### Limitations

Inconsistencies, especially in severe or rare conditions, highlight the challenges of relying solely on social media content and LLMs as stand-alone diagnostic tools. Of note, we did observe that both groups consistently classified few posts as AHC, emphasizing the need for further prompt optimization specific to AHC as well as for human oversight of high-risk outbreaks red flagged by LLMs; and the potential for LLMs to complement, rather than replace, human judgment in assessing the urgency of detected potential outbreaks.

#### Expert Validation of Etiology

##### Overview

For etiology**,** the trained ophthalmologists demonstrated a high specificity in identifying noninfectious causes of conjunctivitis, indicating a strong agreement on what clearly does not constitute a certain type of conjunctivitis based on the posts. This suggests that medical training can result in a consistent ability to rule out certain conditions. However, sensitivity in detecting the specific etiology of infectious conjunctivitis, such as viral and AHC, varied. This variation between the expert ophthalmologists (eg, sensitivities of 0.38 and 0.32 for viral conjunctivitis) aligns with known clinical challenges; for example, studies have shown that even in culture-positive cases of bacterial conjunctivitis, 58% had symptoms of itching (typically associated with allergic causes), and 35% had serous or no discharge (which would indicate to a clinician that the infection is less likely to be bacterial) [73-75]. Despite varied sensitivity for detecting AHC etiology, the sensitivities of 0.71 and 3 out of 4 (too small to reliably estimate sensitivity), in light of the specificity values of 0.99 and 0.96, suggest that LLMs could reliably rule out irrelevant posts and identify some small, early AHC outbreaks (as seen with MD1, with high sensitivity), which, one could make the case, has value from a public health perspective.

##### Limitations

The variation seen between the specialists could stem from individual differences in clinical experience, judgment, or even the subjective interpretation of the social media content’s context and descriptions. This may highlight the nuanced nature of identifying complex conditions from unstructured data, a concept that is also true in clinical settings, often requiring laboratory testing to consistently define the type or etiology [71,72]. They likely also reflect the constraints of social media data, which may lack comprehensive clinical details, affecting the reliability of such data on its own for disease identification. Future studies could conduct targeted social media campaigns to interact with the users posting on social media and survey them on any clinical diagnoses or even collect samples for a laboratory study.

#### Prompt Optimizations

The iterative prompt optimization process yielded modest but consistent improvements in performance, as shown by the gradual increase in correlation coefficient values from prompt 1 to the final prompt. Perhaps, providing broader context and multiple classification tasks in a prompt, rather than multiple simpler prompts asking 1 question at a time, may help the model develop a more nuanced understanding of the content. The structural organization of the prompt—specifically, placing the post content at the end rather than at the beginning—had a meaningful impact on classification accuracy. These results demonstrate that while prompt engineering can enhance LLM performance, the improvements tend to be incremental rather than transformative, with each iteration providing small but measurable gains in accuracy.

### Future Applications of LLM-Based Outbreak Analysis

Our findings suggest that by using social media content and LLMs, we can leverage the assessed characteristics of a large number of known outbreaks to test generalizable hypotheses about epidemics; for example, we might be able to test whether a group of cases have higher (reported; “digital”) severity during outbreaks versus individual nonoutbreak cases. Our approach may provide a new opportunity to leverage information about epidemics in small community settings to ask research questions at even a household epidemic level; for example, can we use information about these microepidemics to predict the emergence of larger, more traditionally detectable epidemics, particularly the severe ones? A future study could collect social media content from the time and location of known infectious conjunctivitis outbreaks with documented severity and primary etiologies and assess the ability of LLMs to characterize these outbreaks, including etiology. Such a study could assess and characterize symptoms as well, if known [76-79]. Finally, with our validated findings of LLMs’ reliable ability to identify mentions of other health conditions, our epidemic detection and characterization approaches could be studied for their potential to reveal early signs of other significant public health concerns from social media content, such as influenza outbreaks, COVID-19, foodborne illness, and contaminated products.

### Limitations of Synthetic Data and Potential Impact on Generalizability

While synthetic data offer advantages in controlling for specific outbreak scenarios and rare events and powering statistical analyses, several limitations may affect the generalizability of our findings to diverse real-world settings. Our synthetic posts do not fully capture the natural language variation, contextual noise (eg, slang and cultural references), complex outbreak dynamics, temporal patterns, user interactions, or misinformation dynamics that characterize real social media discussions across a wide range of users and platforms. While our study provides valuable insights into LLMs’ potential for epidemiological analysis, the performance observed on synthetic data may represent a scenario that might not be fully realized in more complex and noisy real-world settings. These limitations should be carefully considered when interpreting our results, and we emphasize that our synthetic data should be viewed as complementary to, rather than a replacement for, real-world data in developing and validating LLM-based public health surveillance systems. To address these limitations, we recommend that future research pursue extensive validation with diverse real-world data beyond the Twitter/X, forum, and YouTube posts used in this study; develop more sophisticated synthetic data–generation techniques; conduct comparative studies on model performance on synthetic versus real-world data; collaborate with social media platforms to access representative datasets; and incorporate domain expert knowledge to refine synthetic data–generation processes.

### Ethical Implications of AI in Public Health Surveillance

The application of AI in public health surveillance, while promising, raises important ethical considerations that warrant careful attention [80-82]. Key challenges include privacy concerns around social media data use, potential surveillance overreach, algorithmic bias, and the need to balance public health benefits with individual rights. While our study demonstrates LLMs’ potential for enhancing outbreak detection, implementing such systems requires robust safeguards, including strict data anonymization protocols, clear oversight mechanisms, and regular bias assessments. Particular attention must be paid to ensuring that surveillance systems account for potential demographic skews in social media data sources to avoid perpetuating health disparities. Future research should focus not only on advancing technical capabilities but also on establishing governance frameworks that promote responsible AI use in public health surveillance. This includes maintaining clear accountability structures, engaging in ongoing public dialogue, and regularly evaluating the necessity and proportionality of AI-powered surveillance systems to protect both public health and individual privacy.

### Conclusions

#### Key Findings

Our findings suggest that top-performing LLMs can reliably infer the probability and classification of conjunctivitis outbreaks as determined from multiple sources of social media data. This could lead to the ability to better understand outbreak dynamics. This approach could uncover outbreaks that might not yet have been detected or well characterized by traditional clinical or epidemiological reporting datasets or systems.

#### Practical Impact

Moreover, identifying higher-risk severe epidemics through social media monitoring could enable public health authorities to take timely, targeted actions to address severe outbreaks earlier, potentially mitigating their impact.

#### Future Implementation Framework

Our findings lay the groundwork for considering the development of hybrid AI-human surveillance systems where LLMs serve as a near–real-time, initial screening layer for social media content, identifying potential severe outbreak signals for human expert review. This tiered approach could be especially valuable for initially detecting small but significantly severe outbreaks that might be missed by traditional surveillance methods—alerting public health professionals and thus allowing them to focus their expertise and limited time on investigating and responding to the most critical signals. Future research should consider implementing these human-AI workflows, establishing clear escalation protocols, and creating efficient public health surveillance systems that combine automated AI monitoring with expert human judgment.

## Appendix

Multimedia Appendix 1 Additional methodological details.

Multimedia Appendix 2 Boolean query and data.

Multimedia Appendix 3 Human rater training.

Multimedia Appendix 4 Full set of large language models, performance measures, post sources, and human raters (expansion of Table 3).

## Abbreviations

AHC: acute hemorrhagic conjunctivitis
API: application programming interface
BERT: bidirectional encoder representations from transformers
ICC: intraclass correlation coefficient
LLM: large language model

## Data Availability

The primary social media datasets analyzed during this study are not publicly available owing to our terms of use agreement with the Brandwatch platform. However, post IDs and synthetic posts are available from the corresponding author on reasonable request under a data-sharing agreement. The post IDs have also been deposited in the Qualitative Data Repository via the University of California San Francisco.
