# Supplementary material for: Use of Large Language Models to Classify Epidemiological Characteristics in Synthetic and Real-World Social Media Posts About Conjunctivitis Outbreaks: Infodemiology Study
Source: J Med Internet Res. 2025 Jul 2;27:e65226. doi: 10.2196/65226 (PMC12268217; doi:10.2196/65226)
Supplement: Multimedia Appendix 1 [file jmir_v27i1e65226_app1.pdf]

Additional Methodological Details

a) Algorithm For Generating Synthetic Posts

````
```{r, eval=FALSE}
function generateSyntheticTweets(numTweets, probDB, settDB, conjDB, orgDB, sevDB, altProb)
  for i = 1 to numTweets do
    probComponent = randomly select a component from probDB
    settComponent = randomly select a component from settDB
    sevComponent = randomly select a component from sevDB

    if random number < altProb then
      mainPhrase = randomly select a phrase from alternativePhrases
      isAlt = true
    else
      mainPhrase = randomly select a phrase from conjunctivitisRelatedPhrases
      isAlt = false
    end if

    j1, j2, j3, j4, j4a = randomly select joining phrases from joiningPhraseLists

    if isAlt then
      probScore = 5
    else
      probScore = get probability score from probComponent
    end if

    sizeCat = get size category from settComponent
    severity = get severity from sevComponent

    if probScore <= 25 and sizeCat <= 50 and severity is "mild" then
      conjType = randomly select from all conjunctivitis types in conjDB
    else
      conjType = randomly select from non-environmental types in conjDB
    end if

    if conjType is "blank" then
      conjComponent = ""
      j4 = ""
      conjSeverity = "mild"
    else
      j5 = randomly select a joining phrase
      conjComponent = concatenate 2 random components from conjDB where type=conjType,
joined by j5
````
````

```
      conjSeverity = get severity from first selected conjunctivitis component
    end if

    if conjType is "allergic" or "environmental" or "blank" then
      if random number < 1.0 then
        tweet = concatenate(probComponent, j1, settComponent, j2, mainPhrase, j3, conjComponent, j4, sevComponent)
      else
        tweet = concatenate(sevComponent, j4, probComponent, j1, settComponent, j2, mainPhrase, j3, conjComponent)
      end if
      orgComponent = ""
      orgType = null
      orgSeverity = ""
    else if isAlt then
      j5a = randomly select a joining phrase
      tweet = concatenate(probComponent, j1, settComponent, j2, mainPhrase, j3, conjComponent, j4, sevComponent)
      orgComponent = concatenate 2 random non-eye-related components from orgDB, joined by j5a
      orgType = null
      orgSeverity = get severity from selected organism components
    else
      orgType = randomly select an organism type from orgDB
      if orgType is not "blank" then
        j5a = randomly select a joining phrase
        orgComponent = concatenate 2 random components from orgDB where type=orgType, joined by j5a
        if random number < 0.5 then
          tweet = concatenate(probComponent, j1, settComponent, j2, mainPhrase, j3, conjComponent, j4, orgComponent, j4a, sevComponent)
        else
          tweet = concatenate(sevComponent, j4a, probComponent, j1, settComponent, j2, mainPhrase, j3, conjComponent, j4, orgComponent)
        end if
        orgSeverity = get severity from selected organism components
      else
        if random number < 0.5 then
          tweet = concatenate(probComponent, j1, settComponent, j2, mainPhrase, j3, conjComponent, j4, sevComponent)
        else
          tweet = concatenate(sevComponent, j4, probComponent, j1, settComponent, j2, mainPhrase, j3, conjComponent)
        end if
```

```
            orgComponent = ""
            orgSeverity = ""
        end if
    end if

    maxSeverity = max(severity, conjSeverity, orgSeverity)

    store generated tweet and metadata
  end for

  return generated tweets and metadata
end function
```

b) Synthetic Post Generation: Deeper Dive and Bias Mitigation

Here we provide detailed description of the framework, and highlight elements designed to mitigate bias. The system draws from five extensively curated databases containing categorized components: type components (defining outbreak characteristics), organism references (bacterial, viral, and other pathogens), severity indicators (ranging from mild to severe symptoms), credibility markers (from verified medical sources to unconfirmed rumors), and setting descriptions (spanning individual to metropolitan scales). These core databases are supplemented with comprehensive lists of condition-specific terms (e.g., “pink eye”, “conjunctivitis”) and alternative medical conditions (e.g., “flu”, “covid”), along with varied linguistic connectors to ensure natural language flow. While this structured approach enables reproducible generation of posts with known characteristics, it may introduce systematic bias through oversimplification of natural language patterns and cultural expressions, particularly in how different communities discuss health concerns.

The core generation algorithm (genposts) assembles posts through a multi-stage process, randomly selecting and combining elements from each database using sophisticated probabilistic rules. Each generated post receives comprehensive metadata attribution, including unique identifiers, component source information, credibility scores (scaled from 2-95, with lower scores for unverified sources), setting size categories (from individual to metropolitan scale), severity classifications (mild/moderate/strong/extreme), and boolean flags for structural variants. The metadata system captures location characteristics through multiple fields including specific location text, numerical population capacity, and categorical classification of location types. Clinical and epidemiological characteristics are recorded across several dimensions, including outbreak type (allergic, bacterial, viral, environmental), symptom presentations, and pathogen-specific content where applicable. While this systematic generation process enables controlled experimentation and ground-truth validation, it may over-represent well-structured content compared to the more chaotic, organic nature of real social media discussions, potentially biasing our dataset toward idealized representations of outbreak discussions.

To enhance realism and mitigate potential biases, we incorporated diverse linguistic features typical of social media communications, including deliberate misspellings ("seemt", "complinaing"), casual language ("lotta peeps"), and mixed medical-lay terminology. The system generates content across a broad spectrum of credibility levels, from verified medical sources ("doctor confirmed") to unconfirmed rumors ("heard someone say"), and varies symptom descriptions across environmental triggers (e.g., "swimming pool and chlorine"), severity indicators (from "barely noticeable" to "emergency"), and clinical presentations (e.g., "thick, pus-like discharge" for bacterial, "thin, watery discharge" for viral). Population descriptors effectively convey outbreak scope through varied size indicators ("half of the peopl", "the entire dorm wing") and demographic specifics ("all of the kids", "our whole household"). However, despite these efforts to introduce variety, our approach may not fully capture the nuanced ways different cultural, linguistic, and socioeconomic groups discuss health concerns online, potentially under-representing certain communities' expressions and experiences.

The system implements robust quality control measures and internal consistency checks between variables - for example, linking high-credibility sources with larger outbreaks, environmental factors with appropriate settings (e.g., pool-related outbreaks in recreational facilities), and symptom patterns with their associated clinical presentations. Credibility scores are distributed across three main bands: low credibility (2-10) for unverified claims, medium credibility (40-70) for general outbreak discussions, and high credibility (71-95) for authoritative sources. Setting classifications span from "location tiny" (4-5 person capacity) to "location very large" (10,000+ capacity), enabling analysis across different scales of potential exposure and population density. While these logical relationships improve post coherence and realism, they may oversimplify the complex, sometimes contradictory nature of real outbreak discussions on social media. Our bias mitigation efforts included ensuring geographical diversity in locations, balanced gender representation in personal narratives, inclusion of posts in multiple languages (proportional to global language usage), and varied literacy levels in post construction.

Despite the extensive efforts to create diverse and realistic posts, we acknowledge several significant potential sources of bias in our synthetic data generation process: over-representation of clear, well-structured posts compared to the more ambiguous and messy nature of real social media content; potential under-representation of cultural or regional specific expressions of health concerns, particularly from marginalized or underserved communities; simplified representation of outbreak dynamics compared to the complex social, environmental, and biological interactions in real-world outbreaks; and possible over-emphasis on textual content, neglecting the rich multimedia aspects of modern social media such as images, videos, and interactive elements. The synthetic post generator produces output in CSV format with comprehensive metadata, facilitating subsequent analysis and classification tasks, but this structured format itself may impose artificial constraints on the natural variability of social media communications.

c) Data Extraction Prompt

DATA EXTRACTION PROMPT: "secprompt" : "Please use the information provided to fill out the XML form fields indicated: <location> </location> <probability> </probability>

<number_affected> </number_affected> <type_of_outbreak> </type_of_outbreak> <cause></cause> <severity></severity> <symptoms> </symptoms> <healthcondition> </healthcondition> <explanation> </explanation>. Include the answer between the matching tags; for instance, a probability of X% would be indicated as <probability>X%</probability>. Please do not change the format of probabilities given as percentages; leave percentages in their original form. Express number_affected in the form of a Hindu-Arabic numeral; do not use words. Given this information: \"{}\""
