# Supplementary material for: Use of Large Language Models to Classify Epidemiological Characteristics in Synthetic and Real-World Social Media Posts About Conjunctivitis Outbreaks: Infodemiology Study
Source: J Med Internet Res. 2025 Jul 2;27:e65226. doi: 10.2196/65226 (PMC12268217; doi:10.2196/65226)
Supplement: Multimedia Appendix 2 [file jmir_v27i1e65226_app2.pdf]

Boolean Query and Data

The details of the Twitter/X and forums query are as shown below (not shown: additional exclusion of terms to remove posts about animals, obscenities, artistic or literary references, celebrities and politicians):

“(("aankh aana" OR "azoumounou" OR "Bindehautentzündung" OR "bindhinneinflammation" OR "bindvliesontsteking" OR "congiuntivite" OR "conjonctivite" OR "conjunctivită" OR "conjunctivitis" OR "conjuntivite" OR "conjuntivitis" OR "je woz" OR "konjonktivit" OR "konjuktiviti" OR "konjuktivitis" OR "konjunktivitis" OR "konjunktivīts" OR "konjunktivitt" OR "konjunktyvitas" OR "kötőhártya-gyulladás" OR "œil rose" OR "pinkeye" OR "pink eye" OR "sidekalvontulehdus" OR "sidekestapõletik" OR "Viêm kết mạc" OR "viem ket mac" OR "zánětspojivek" OR "zapaleniespojówek" OR "zápalspojiviek" OR " конјуктивит " OR " конъюнктивит " OR " конюнктивит " OR " التهابالملتحمة" OR " آنکھکالالہوجانا" OR " आखका ला लहो जा न" OR " ਅੰਖਦਾ ਲਾ ਲਹੋਜਾ ਣਾ" OR " ตาแดง " OR "결막염" OR "結膜炎 " OR "結膜炎 " OR "结膜炎"))

AND

(our OR my OR hers OR his OR her OR him OR their OR he OR she OR he OR we)

AND

((((outbreak OR epidemic OR "going around" OR "spreading" OR cancelled OR closed OR postponed OR "the entire" OR "everybody" OR "everyone" OR "my entire" OR "the whole") NEAR/5 (has OR got OR gotten OR "coming down with" OR getting OR sick OR spreading)) NEAR/15 (pollen OR smoke OR swimming OR pool OR pollution OR fires OR pus OR "stuck shut" OR green OR yellow OR swollen OR edema OR itch OR burn*))*

OR

(((outbreak OR epidemic OR "going around" OR "spreading" OR cancelled OR closed OR postponed OR "the entire" OR "everybody" OR "everyone" OR "my entire" OR "the whole") NEAR/5 (has OR got OR gotten OR "coming down with" OR getting OR sick OR spreading)) NEAR/5 (bacteri OR virus OR viral OR allergic)))*

The YouTube query was similar but broader (not shown: additional exclusion of terms to remove posts about animals, obscenities, artistic or literary references, celebrities and politicians):

“(("aankh aana" OR "azoumounou" OR "Bindehautentzündung" OR "bindhinneinflammation" OR "bindvliesontsteking" OR "congiuntivite" OR "conjonctivite" OR "conjunctivită" OR "conjunctivitis" OR "conjuntivite" OR "conjuntivitis" OR "je woz" OR "konjonktivit" OR "konjuktiviti" OR "konjuktivitis" OR "konjunktivitis" OR "konjunktivīts" OR "konjunktivitt" OR "konjunktyvitas" OR "kötőhártya-gyulladás" OR "œil rose" OR "pinkeye" OR "pink eye" OR "sidekalvontulehdus" OR "sidekestapõletik" OR "Viêm kết mạc" OR "viem ket mac" OR "zánětspojivek" OR "zapaleniespojówek" OR "zápalspojiviek" OR " конјуктивит " OR " конъюнктивит " OR " конюнктивит " OR " التهابالملتحمة" OR " آنکھکالالہوجانا" OR " आखका ला लहो जा न" OR " ਅੱਖਦਾ ਲਾ ਲਹੋਜਾ ਣਾ" OR " ตาแดง " OR "결막염 " OR "結膜炎 " OR "結膜炎 " OR "结膜炎" OR conjunc?iv?t?s OR "eye infection" OR #conjunctivitis OR #pinkeye)
NEAR/15 (outbreak OR has OR got OR have OR had OR gotten OR "coming down with" OR getting OR sick OR spreading OR house OR school OR work OR classroom OR school OR preschool OR daycare OR kids OR house OR "going around"))

NEAR/15 (outbreak OR has OR got OR have OR had OR gotten OR ""coming down with"" OR getting OR sick OR spreading OR house OR school OR work OR classroom OR school OR preschool OR daycare OR kids OR house OR ""going around""))

Table S1 Data Distribution for Prompt Optimization and Validation

Data Source	Total Posts	LLM Prompt Optimization	LLM Validation
**Synthetic**	>1,402	< 250 per iteration round	1,152
**Twitter/X**	370	0	370
**forums**	290	0	290
**YouTube**	956	0	956
