# Supplementary material for: Use of Large Language Models to Classify Epidemiological Characteristics in Synthetic and Real-World Social Media Posts About Conjunctivitis Outbreaks: Infodemiology Study
Source: J Med Internet Res. 2025 Jul 2;27:e65226. doi: 10.2196/65226 (PMC12268217; doi:10.2196/65226)
Supplement: Multimedia Appendix 3 [file jmir_v27i1e65226_app3.pdf]

Human Rater Training

a) Human Rater Calibration and Alignment Sessions: All human graders took part in a calibration and alignment session conducted via Zoom with one of our study team members, and using Qualtrics surveys (Qualtrics International Inc). Qualtrics (Qualtrics International Inc., Provo, UT) is a web-based survey and data collection platform widely used in academic and market research. In our study, we utilized Qualtrics to create and administer standardized surveys for our human graders. These surveys were designed to: (a) Present social media posts in a consistent format to all graders; (b) Collect graders' assessments of epidemiological features (e.g., outbreak probability, size, type, severity) in a structured manner; (c) Ensure uniformity in data collection across different graders and grading sessions; (d) Facilitate easy export of collected data for subsequent analysis. The use of Qualtrics allowed us to efficiently manage the human validation process, minimize data entry errors, and standardize the grading experience across all participants. Each survey question corresponded to a specific epidemiological feature we aimed to evaluate, mirroring the structure of our LLM prompts to enable direct comparisons between human and LLM assessments.

This Zoom session aimed to familiarize graders with the Qualtrics survey, task, ensure alignment and consistency in their assessments, and introduce them to the nuances of social media language. The session data included in the Qualtrics survey consisted of two distinct sets, each containing 20 posts (not all posts were necessarily used in the training). During the first part of the session, for each section (outbreak size, type, severity, etc.) human graders collaboratively reviewed the original prompts provided to the LLMs and then reviewed instructions provided to them on how review each post and assign classifications and/or probability scores using the Qualtrics survey. This was followed by a facilitated group discussion to address discrepancies in classifications and familiarize graders with social media communication styles, including the use of hashtags and sarcasm. For the second session, graders independently reviewed the posts and assigned classifications and probability scores using the Qualtrics survey. A brief discussion was then conducted to ensure consensus and further refine their classification skills.

b) Qualtrics Instructions that Human Graders Were Provided, and Additional Etiological Cause GPT-4 Prompt

1) Outbreak Probability

The instructions in the Qualtrics survey were: *"Here is a text portion of our request "prompt" that we had provided to the LLM to get its responses: [LLM PROMPT WAS HERE]. Below, is a modified version of the same request for you: Please read each snippet in blue and then based upon that content, indicate* ***how certain are you that this snippet is about a multiperson outbreak of pink eye occurring at the time the snippet was posted****? For each row,* ***you can hover your mouse over the "hover here for full instructions" to review the entire LLM prompt instructions again,*** *including when to assign a 0% or other very low likelihood (for example if it you are certain it is fictional or a rumor proven incorrect etc). Please try the hover function to be sure you can see the pop-up of the full portion of the prompt instructions to the LLMs."*

2) Outbreak Severity, Size, Type

The instructions in the Qualtrics survey were: *"Here is a text portion of our request "prompt" that we had provided to the LLM to get its responses: [LLM PROMPT WAS HERE]. Below, in each row we will provide a modified version of the above prompt as requests for you. For each row, we will ask you to read the blue snippet and then estimate the info about the mentioned conjunctivitis cases: Severity, Number of Cases, Type of Cases. Here are guidelines and definitions to please read before proceeding (these will be briefly described in each row for your convenience): 1) Severity of these cases on health: "**NOT SPECIFIED**" (too hard to tell severity), "**MILD**" (not significant), "**MODERATE"** (has some impact on health),"**STRONG**" (has serious health impact), "**SEVERE**" (life-threatening). 2) Number of people affected: Best guess based in part on the estimated population of the location and how many of them might be affected. Type the NUMBER(integer)--or--type "N" for "not defined". Examples: 2 | 5,500 | N . 3) Type of conjunctivitis cases: "**NOT SPECIFIED**" (select this if it's too hard to feel very confident guessing the type of conjunctivitis). "**ALLERGIC**" (select this if you think allergic conjunctivitis for example if it's about allergy season, or symptoms of allergy or pollen). "**INFECTIOUS**" (select this if you think it may be viral or bacterial conjunctivitis, but NOT AHC). "**ENVIRONMENTAL**" (select this if you think the conjunctivitis is from swimming pools, pollution, toxic spills, smoke, wildfires, drug-usage). "**AHC-INFECTIOUS**" (select this if you suspect is may be AHC -- acute hemorrhagic conjunctivitis, also known epidemic keratoconjunctivitis, hemorrhagic conjunctivitis -- is very severe and typically includes extremely red, bloody or bleeding eyes, vision loss and other severe symptoms)."*

3) Health Conditions:

The instructions in the Qualtrics survey were: *"Here is a text portion of our request "prompt" that we had provided to the LLM to get its responses: [LLM PROMPT WAS HERE]. For every snippet provide the following: tell us the health condition(s) or disease(s) being discussed (e.g. conjunctivitis, flu, broken leg, etc.)" Below is our request for you, a modified version of the prompt above. **For each row, read the blue snippet and** based upon that content, please provide the following: Tell us **what health condition(s) or disease(s) are being discussed (e.g. conjunctivitis, flu, broken leg, etc.)**? (please DO provide health condition/disease names, even if you suspect this is not about a real case of that condition). Please list health conditions (e.g. conjunctivitis, flu, broken leg, etc) but do not list individual symptoms (e.g. itchy eyes, fever, pain) unless you feel that that symptom is a known health condition."*.

4) Specialist Validation of Conjunctivitis Etiological cause

The additional GPT-4 prompt for the conjunctivitis etiological cause included: "...*Cause: Please assign an infectious or non-infectious cause, choosing one of the following etiology categories: 1. \"NOT INFECTIOUS\" (select this if you think it seems not infectious), 2. \"INFECTIOUS: UNSPECIFIED\" (select this if it's too hard to feel very confident guessing the type of organism but it seems infectious), 3. \"BACTERIAL\" (select this if you think it may be bacterial conjunctivitis), 4. \"VIRAL: UNSPECIFIED\" (select this if you think it may be viral conjunctivitis but not COVID-19 or AHC), 5. \"VIRAL: COVID-19\" (select this if you think it may be viral COVID-19 conjunctivitis more than other forms of viral conjunctivitis), 6. \"VIRAL: AHC\" (select this if*

you think it may be viral AHC conjunctivitis more than other forms of viral conjunctivitis)....”

The instructions in the Qualtrics survey for the two trained surface disease practicing ophthalmologists to assess the conjunctivitis etiological cause included: ***“...For each row, we will ask you to read the blue snippet and then estimate the conjunctivitis cases: Severity, Type of Cases and Etiology of Cases.*** *Here are guidelines and definitions to please read before proceeding (these will be briefly described in each row for your convenience)...**Etiology of cases: "NOT INFECTIOUS"** (select this if you think it seems not infectious); **"INFECTIOUS: UNSPECIFIED"** (select this if it's too hard to feel very confident guessing the type of organism but it seems infectious); **"BACTERIAL"** (select this if you think it may be bacterial conjunctivitis); **"VIRAL: UNSPECIFIED"** (select this if you think it may be viral conjunctivitis but not COVID-19 or AHC); **"VIRAL: COVID-19"** (select this if you think it may be viral COVID-19 conjunctivitis more than other forms of viral conjunctivitis); **"VIRAL: AHC"** (select this if you think it may be viral AHC conjunctivitis more than other forms of viral conjunctivitis). Once you become familiar with the definitions above, please now proceed with each row below".*
