# Supplementary material for: Use of Large Language Models to Classify Epidemiological Characteristics in Synthetic and Real-World Social Media Posts About Conjunctivitis Outbreaks: Infodemiology Study
Source: J Med Internet Res. 2025 Jul 2;27:e65226. doi: 10.2196/65226 (PMC12268217; doi:10.2196/65226)
Supplement: Multimedia Appendix 4 [file jmir_v27i1e65226_app4.pdf]

Full Set of LLMs, Performance Measures, Post Sources and Graders (Expansion of Table 3)

Supplement Table S1. Inter-rater Reliability Analysis of GPT-4 and Human Classifications

	All Raters	Non-Experts Only	MD Experts Only	MD vs. Non-Experts	GPT-4 vs. Non-Experts	GPT-4 vs. MD Experts
**A.**						
**Outbreak Probability**	0.65 (0.53, 0.75)	0.76 (0.65-0.84)	n/a	n/a	0.63 (0.47-0.75)	n/a
**Outbreak Severity**	0.40 (0.18-0.60)	0.18 (-0.08-0.50)	0.38 (0.05-0.61)	0.52 (0.30-0.68)	0.59 (0.40-0.74)	0.69 (0.56-0.79)
**Outbreak Size**	0.62 (0.45-0.75)	0.77 (0.59-0.86)	n/a	n/a	0.64 (0.40-0.79)	n/a
**B.**						
**Outbreak Types:**						
**Infectious**	0.43 (0.31-0.54)	0.58 (0.39-0.74)	0.25 (0.01-0.46)	n/a	n/a	n/a
**Allergic**	0.78 (0.67-0.88)	0.84 (0.66-0.97)	0.69 (0.41-0.88)	n/a	n/a	n/a
**AHC**	0.37 (0.24-0.45)	-0.04 (-0.07-0.01)	-0.04 (-0.07-0.01)	n/a	n/a	n/a
**Environmental**	0.24 (0.11-0.35)	0.29 (-0.06-0.64)	-0.01 (-0.02-0.01)	n/a	n/a	n/a
**Health Conditions:**						
**Conjunctivitis**	0.94 (0.84-1.0)	0.94 (0.84-1.0)	n/a	n/a	n/a	n/a
**COVID-19**	0.88 (0.61-1.0)	0.83 (0.49-1.0)	n/a	n/a	n/a	n/a
**Influenza**	0.82 (0.53-1.0)	0.83 (0.50-1.0)	n/a	n/a	n/a	n/a
**Intestinal Flu**	0.46 (-0.02-0.79)	0.48 (-0.05-1.0)	n/a	n/a	n/a	n/a
**Croup**	1.0 (1.0-1.0)	1.0 (1.0-1.0)	n/a	n/a	n/a	n/a
**Lice**	0.95 (0.81-1.0)	-	n/a	n/a	n/a	n/a
**Broken Leg**	1.0 (1.0-1.0)	1.0 (1.0-1.0)	n/a	n/a	n/a	n/a

Supplement Table S1. Inter-rater Reliability Analysis of LLM and Human Classifications. This table presents the agreement between different rater groups using Intraclass Correlation Coefficient (ICC) for continuous measures (**Panel A**) and Fleiss' Kappa (κ) for categorical classifications (**Panel B**). Panel A shows ICC values (95% CI) for continuous measures including outbreak probability, severity and size across different rater groups. Panel B shows Fleiss' Kappa values (95% CI) for categorical classifications of outbreak types and health conditions. Empty cells (-) indicate insufficient data for reliable calculation. Cells where assessments were not done by humans or LLMs are indicated with "n/a" since that specific comparison was not applicable. ICC values range from 0 to 1, where values <0.40 indicate poor agreement, 0.40-0.75 indicate fair to good agreement, and >0.75 indicate excellent agreement. Kappa values follow similar interpretation ranges, though negative values are possible and indicate agreement worse than expected by chance.

Table S2. Validating LLMs against known values – all 7 LLMs shown

LLM Characterizations	GPT 4	Sonnet 3	Mixtral 8x22B	Opus	LlaMa 3 70B	GPT 4o	GPT 3.5
**Outbreak (OB) Probability**	0.73 (0.70, 0.76)	0.73 (0.70, 0.76)	0.77 (0.74, 0.79)	0.72 (0.69, 0.75)	0.71 (0.67, 0.74)	0.71 (0.68, 0.74)	0.71 (0.68, 0.74)
**OB Severity of Cases**	0.82 (0.79, 0.85)	0.81 (0.78, 0.84)	0.80 (0.77, 0.83)	0.91 (0.89, 0.93)	0.86 (0.83, 0.88)	0.86 (0.83, 0.88)	0.78 (0.75, 0.81)
**OB Size (# of Cases)**	0.54 (0.47, 0.59)	0.45 (0.39, 0.52)	0.37 (0.30, 0.43)	0.67 (0.63, 0.71	0.45 (0.37, 0.53)	0.56 (0.51, 0.61)	0.63 (0.52, 0.72)
OUTBREAK TYPE							
**Infectious, Sensitivity**	1.0 (448/450)	0.99 (445/450)	1.0 (450/450)	0.99 (444/450)	0.98 (443/450)	0.99 (444/450)	1.0 (449/450)
**Infectious, Specificity**	0.68 (191/281)	0.62 (174/281)	0.64 (181/281)	0.52 (146/281)	0.42 (117/281)	0.66 (185/281)	0.33 (93/281)
**Allergic, Sensitivity**	0.57 (87/153)	0.41 (63/153)	0.54 (82/153)	0.37 (57/153)	0.12 (18/153)	0.52 (79/153)	0.34 (52/153)

LLM Characterizations	GPT 4	Sonnet 3	Mixtral 8x22B	Opus	LlaMa 3 70B	GPT 4o	GPT 3.5
Allergic, Specificity	1.0 (578/578)	1.0 (578/578)	1.0 (578/578)	1.0 (578/578)	1.0 (578/578)	1.0 (578/578)	1.0 (578/578)
AHC, Sensitivity	0.56 (83/148)	0.28 (42/148)	0.52 (77/148)	0.52 (77/148)	0.56 (83/148)	0.66 (97/148)	0.034 (5/148)
AHC, Specificity	1.0 (581/583)	1.0 (581/583)	0.99 (580/583)	1.0 (583/583)	0.99 (580/583)	0.98 (571/583)	1.0 (583/583)
Environ., Sensitivity	1.0 (103/103)	1.0 (103/103)	0.95 (98/103)	0.96 (99/103)	0.85 (88/103)	1.0 (103/103)	0.35 (36/103)
Environ., Specificity	0.99 (624/628)	0.97 (612/628)	0.98 (618/628)	1.0 (628/628)	0.99 (620/628)	1.0 (625/628)	1.0 (626/628)
HEALTH CONDITIONS							
Conjunct., Sensitivity	0.85 (816/961)	0.87 (839/961)	0.95 (910/961)	0.85 (819/961)	0.96 (922/961)	0.83 (801/961)	0.75 (718/961)
Conjunct., Specificity	0.89 (170/191)	0.89 (170/191)	0.84 (160/191)	0.84 (160/191)	0.77 (148/191)	0.91 (173/191)	0.83 (158/191)
COVID-19, Sensitivity	1.0 (9/9)	1.0 (9/9)	1.0 (9/9)	1.0 (9/9)	1.0 (9/9)	1.0 (9/9)	0.78 (7/9)
COVID-19, Specificity	0.99 (1131/1143)	0.99 (1133/1143)	0.99 (1135/1143)	0.99 (1129/1143)	0.99 (1133/1143)	0.99 (1136/1143)	1.0 (1139/1143)
Influenza, Sensitivity	0.96 (46/48)	0.94 (45/48)	0.96 (46/48)	1.0 (48/48)	0.96 (46/48)	0.88 (42/48)	0.79 (38/48)
Influenza, Specificity	1.0 (1102/1104)	0.99 (1098/1104)	1.0 (1104/1104)	1.0 (1102/1104)	1.0 (1101/1104)	1.0 (1104/1104)	1.0 (1104/1104)
Int. Flu, Sensitivity	0.33 (48/145)	0.27 (39/145)	0.36 (52/145)	0.31 (45/145)	0.26 (38/145)	0.27 (39/145)	0.15 (22/145)
Int. Flu, Specificity	0.98 (990/1007)	0.99 (1001/1007)	0.99 (997/1007)	0.98 (990/1007)	0.99 (999/1007)	0.99 (1001/1007)	1.0 (1005/1007)
Croup, Sensitivity	0.89 (17/19)	0.95 (18/19)	0.74 (14/19)	0.95 (18/19)	0.79 (15/19)	0.74 (14/19)	0.42 (8/19)
Croup, Specificity	1.0 (1133/1133)	1.0 (1133/1133)	1.0 (1133/1133)	1.0 (1133/1133)	1.0 (1133/1133)	1.0 (1133/1133)	1.0 (1133/1133)
Lice, Sensitivity	0.76 (19/25)	0.84 (21/25)	0.56 (14/25)	0.80 (20/25)	0.68 (17/25)	0.60 (15/25)	0.12 (3/25)
Lice, Specificity	1.0 (1127/1127)	1.0 (1127/1127)	1.0 (1127/1127)	1.0 (1127/1127)	1.0 (1127/1127)	1.0 (1127/1127)	1.0 (1127/1127)
Leg Break, Sensitivity	0.88 (15/17)	0.65 (11/17)	0.65 (11/17)	0.24 (4/17)	0.53 (9/17)	0.53 (9/17)	0.0 (0/17)
Leg Break, Specificity	1.0 (1134/1135)	1.0 (1134/1135)	1.0 (1135/1135)	1.0 (1135/1135)	1.0 (1135/1135)	1.0 (1134/1135)	1.0 (1135/1135)

Supplement Table S3. Calibration and Reliability of Model Assessments – all 7 LLMs shown

Performance Measure	GPT 4	Sonnet 3	Mixtral 8x22B	Opus	LlaMa 3 70B	GPT 4o	GPT 3.5
Bias	8.8	11.5	20.6	17.4	14.1	14.3	22.2
Calibration Error	16.6	17.7	24.8	23.7	19.6	23.8	25.7
Resolution	25.0	25.3	23.6	23.5	22.5	24.2	20.8
Reliability	0.643	0.657	0.620	0.602	0.615	0.576	0.598
RMSE	26.1	26.3	33.6	32.0	30.4	31.0	36.3

Supplement Table S4. - Twitter/X posts, Comparative Validations of 6 LLMs vs. GPT-4

LLM Characterizations	Sonnet	Mixtral 8x22b	Opus	LlaMa 3	GPT 4o	GPT 3.5
Outbreak (OB) Probability	0.82 (0.76, 0.87)	0.74 (0.67, 0.79)	0.73 (0.63, 0.80)	0.38 (0.11, 0.60)	0.75 (0.65, 0.83)	0.57 (0.48, 0.66)
OB Severity of Cases	0.99 (0.96, 1.0)	1.0 (0.98, 1.0)	1.0 (0.98, 1.0)	1.0 (0.98, 1.0)	1.0 (0.98, 1.0)	0.98 (0.95, 1.0)
OB Size (# of Cases)	0.85 (0.79, 0.90)	0.84 (0.78, 0.88)	0.74 (0.65, 0.81)	0.71 (0.60, 0.79)	0.73 (0.66, 0.80)	0.13 (-0.19, 0.43)
OUTBREAK TYPE						
Infectious, Sensitivity	0.98 (235/241)	0.96 (232/241)	0.95 (228/241)	0.88 (213/241)	0.93 (224/241)	0.71 (171/241)
Infectious, Specificity	0.57 (4/7)	0.29 (2/7)	0.43 (3/7)	0.71 (5/7)	0.57 (4/7)	0.57 (4/7)
Allergic, Sensitivity	(1/1)	(1/1)	(1/1)	(1/1)	(1/1)	(0/1)
Allergic, Specificity	1.0 (246/247)	1.0 (247/247)	1.0 (247/247)	1.0 (247/247)	1.0 (247/247)	1.0 (247/247)
AHC, Sensitivity	(0/0)	(0/0)	(0/0)	(0/0)	(0/0)	(0/0)
AHC, Specificity	1.0 (248/248)	1.0 (248/248)	1.0 (248/248)	1.0 (248/248)	1.0 (248/248)	1.0 (248/248)
Environ., Sensitivity	(3/5)	(1/5)	(1/5)	(1/5)	(1/5)	(0/5)
Environ., Specificity	0.98 (239/243)	1.0 (243/243)	1.0 (243/243)	0.99 (241/243)	1.0 (242/243)	1.0 (243/243)
HEALTH CONDITIONS						
Conjunct., Sensitivity	0.94 (333/354)	0.95 (335/354)	0.94 (332/354)	0.97 (343/354)	0.86 (306/354)	0.57 (203/354)
Conjunct., Specificity	0.44 (7/16)	0.50 (8/16)	0.62 (10/16)	0.44 (7/16)	0.88 (14/16)	0.94 (15/16)
COVID-19, Sensitivity	0.81 (21/26)	0.50 (13/26)	0.77 (20/26)	0.69 (18/26)	0.69 (18/26)	0.50 (13/26)
COVID-19, Specificity	0.98 (338/344)	0.99 (340/344)	0.95 (328/344)	0.99 (339/344)	0.98 (336/344)	0.99 (339/344)
Influenza, Sensitivity	0.68 (13/19)	0.63 (12/19)	0.53 (10/19)	0.58 (11/19)	0.89 (17/19)	0.53 (10/19)
Influenza, Specificity	0.98 (344/351)	0.98 (345/351)	0.98 (345/351)	0.99 (346/351)	0.98 (343/351)	0.99 (346/351)
Int. Flu, Sensitivity	0.89 (16/18)	0.72 (13/18)	0.61 (11/18)	0.61 (11/18)	0.89 (16/18)	0.28 (5/18)
Int. Flu, Specificity	0.99 (348/352)	0.99 (348/352)	0.99 (347/352)	0.99 (348/352)	0.99 (349/352)	0.99 (348/352)
Croup, Sensitivity	(2/2)	(2/2)	(2/2)	(1/2)	(2/2)	(0/2)
Croup, Specificity	1.0 (368/368)	1.0 (368/368)	1.0 (368/368)	1.0 (368/368)	1.0 (368/368)	1.0 (368/368)
Lice, Sensitivity	(1/1)	(0/1)	(0/1)	(0/1)	(0/1)	(0/1)
Lice, Specificity	0.99 (367/369)	0.99 (365/369)	0.99 (366/369)	1.0 (368/369)	1.0 (368/369)	1.0 (369/369)
Leg Break, Sensitivity	(0/0)	(0/0)	(0/0)	(0/0)	(0/0)	(0/0)
Leg Break, Specificity	1.0 (370/370)	1.0 (370/370)	1.0 (370/370)	1.0 (370/370)	1.0 (370/370)	1.0 (370/370)

Supplement Table S5. - Forum posts, Comparative Validations of 6 LLMs vs. GPT-4

LLM Characterizations	Sonnet	Mixtral 8x22b	Opus	LlaMa 3	GPT 4o	GPT 3.5
Outbreak (OB) Probability	0.93 (0.90, 0.95)	0.82 (0.77, 0.86)	0.79 (0.71, 0.85)	0.73 (0.59, 0.82)	0.56 (0.44, 0.67)	0.50 (0.39, 0.60)
OB Severity of Cases	1.0 (0.97, 1.0)	0.99 (0.95, 1.0)	1.0 (0.96, 1.0)	1.0 (0.97, 1.0)	0.98 (0.92, 1.0)	0.98 (0.93, 1.0)
OB Size (# of Cases)	0.95 (0.92, 0.97)	0.82 (0.72, 0.89)	0.64 (0.46, 0.77)	0.84 (0.74, 0.90)	0.36 (0.14, 0.55)	0.69 (0.44, 0.84)

LLM Characterizations	Sonnet	Mixtral 8x22b	Opus	LlaMa 3	GPT 4o	GPT 3.5
OUTBREAK TYPE						
**Infectious, Sensitivity**	0.99 (100/101)	1.0 (101/101)	0.92 (93/101)	0.92 (93/101)	0.71 (72/101)	0.89 (90/101)
**Infectious, Specificity**	0.88 (7/8)	0.75 (6/8)	0.88 (7/8)	0.62 (5/8)	0.88 (7/8)	0.50 (4/8)
**Allergic, Sensitivity**	(0/0)	(0/0)	(0/0)	(0/0)	(0/0)	(0/0)
**Allergic, Specificity**	1.0 (109/109)	1.0 (109/109)	0.99 (108/109)	1.0 (109/109)	0.99 (108/109)	1.0 (109/109)
**AHC, Sensitivity**	(0/0)	(0/0)	(0/0)	(0/0)	(0/0)	(0/0)
**AHC, Specificity**	1.0 (109/109)	1.0 (109/109)	1.0 (109/109)	0.99 (108/109)	1.0 (109/109)	1.0 (109/109)
**Environ., Sensitivity**	0.88 (7/8)	0.75 (6/8)	0.75 (6/8)	0.62 (5/8)	0.88 (7/8)	0.38 (3/8)
**Environ., Specificity**	1.0 (101/101)	1.0 (101/101)	1.0 (101/101)	0.99 (100/101)	0.97 (98/101)	1.0 (101/101)
HEALTH CONDITIONS						
**Conjunct., Sensitivity**	0.95 (219/231)	0.91 (210/231)	0.82 (189/231)	0.95 (220/231)	0.80 (184/231)	0.65 (151/231)
**Conjunct., Specificity**	0.71 (42/59)	0.75 (44/59)	0.90 (53/59)	0.68 (40/59)	0.58 (34/59)	0.86 (51/59)
**COVID-19, Sensitivity**	0.83 (29/35)	0.66 (23/35)	0.80 (28/35)	0.80 (28/35)	0.43 (15/35)	0.54 (19/35)
**COVID-19, Specificity**	0.96 (246/255)	0.98 (249/255)	0.95 (243/255)	0.98 (249/255)	0.91 (233/255)	0.99 (253/255)
**Influenza, Sensitivity**	0.60 (6/10)	0.70 (7/10)	0.60 (6/10)	0.50 (5/10)	0.50 (5/10)	0.30 (3/10)
**Influenza, Specificity**	0.97 (272/280)	0.99 (277/280)	0.99 (276/280)	0.99 (276/280)	0.99 (277/280)	1.0 (280/280)
**Int. Flu, Sensitivity**	0.50 (4/8)	0.62 (5/8)	0.75 (6/8)	0.38 (3/8)	0.50 (4/8)	0.12 (1/8)
**Int. Flu, Specificity**	1.0 (281/282)	0.99 (280/282)	0.98 (276/282)	1.0 (282/282)	0.99 (279/282)	0.99 (279/282)
**Croup, Sensitivity**	(0/0)	(0/0)	(0/0)	(0/0)	(0/0)	(0/0)
**Croup, Specificity**	1.0 (290/290)	1.0 (290/290)	1.0 (290/290)	1.0 (290/290)	1.0 (290/290)	1.0 (290/290)
**Lice, Sensitivity**	(0/0)	(0/0)	(0/0)	(0/0)	(0/0)	(0/0)
**Lice, Specificity**	1.0 (290/290)	1.0 (290/290)	1.0 (290/290)	1.0 (290/290)	1.0 (290/290)	1.0 (290/290)
**Leg Break, Sensitivity**	(0/0)	(0/0)	(0/0)	(0/0)	(0/0)	(0/0)
**Leg Break, Specificity**	1.0 (290/290)	1.0 (290/290)	1.0 (290/290)	1.0 (290/290)	1.0 (290/290)	1.0 (290/290)

Supplement Table S6. - YouTube posts, Comparative Validations of 4 LLMs vs. GPT-4

LLM Characterizations	Mixtral 8x22b	Opus	GPT 4o	Qwen Max
Outbreak (OB) Likelihood	0.75 (0.71, 0.78)	0.66 (0.57, 0.73)	0.36 (0.26, 0.46)	0.77 (0.74, 0.80)
OB Severity of Cases Fuzzy	1.0 (0.98, 1.0)	1.0 (0.98, 1.0)	1.0 (0.98, 1.0)	1.0 (0.99, 1.0)
OB Size (# of Cases)	0.54 (0.44, 0.62)	0.18 (0.055, 0.30)	0.16 (0.037, 0.28)	0.17 (0.043, 0.29)
OUTBREAK TYPE				
Infectious, Sensitivity	0.99 (231/234)	0.94 (219/234)	0.87 (203/234)	0.97 (228/234)
Infectious, Specificity	0.25 (4/16)	0.19 (3/16)	0.31 (5/16)	0.31 (5/16)
Allergic, Sensitivity	(0/1)	(0/1)	(1/1)	(0/1)
Allergic, Specificity	1.0 (248/249)	1.0 (249/249)	1.0 (249/249)	1.0 (249/249)
AHC, Sensitivity	(0/0)	(0/0)	(0/0)	(0/0)
AHC, Specificity	1.0 (250/250)	1.0 (250/250)	1.0 (250/250)	1.0 (249/250)
Environ., Sensitivity	(3/4)	(1/4)	(2/4)	(4/4)
Environ., Specificity	0.98 (242/246)	1.0 (246/246)	0.99 (244/246)	0.98 (241/246)

Supplement Table S7. Comparing Human Validation to GPT-4 Insights, all 4 Human Graders

LLM Characterizations	Non-Expert 1	Non-Expert 2	MD Expert 1	MD Expert 2
**Outbreak (OB) Probability**	0.59 (0.41, 0.72)	0.62 (0.45, 0.74)	n/a	n/a
**OB Severity of Cases**	0.73 (0.59, 0.83)	0.78 (0.65, 0.86)	0.99 (0.93, 1.0)	1.0 (0.96, 1.0)
**OB Size (# of Cases)**	0.98 (0.91, 1.0)	0.98 (0.87, 1.0)	n/a	n/a
OUTBREAK TYPE				
**Infectious, Sensitivity**	1.0 (57/57)	0.98 (47/48)	0.91 (50/55)	0.84 (66/79)
**Infectious, Specificity**	0.68 (21/31)	0.50 (20/40)	0.48 (16/33)	0.89 (8/9)
**Allergic, Sensitivity**	0.93 (13/14)	0.88 (14/16)	0.92 (12/13)	0.89 (8/9)
**Allergic, Specificity**	0.97 (72/74)	0.99 (71/72)	0.96 (72/75)	0.91 (72/79)
**AHC, Sensitivity**	0.57 (4/7)	(0/0)	0.57 (4/7)	(0/0)
**AHC, Specificity**	0.99 (80/81)	0.94 (83/88)	0.99 (80/81)	0.94 (83/88)
**Environ., Sensitivity**	0.67 (6/9)	(1/3)	(1/1)	(0/0)
**Environ., Specificity**	0.99 (78/79)	0.93 (79/85)	0.93 (81/87)	0.92 (81/88)
HEALTH CONDITIONS				
**Conjunct., Sensitivity**	0.88 (15/17)	0.88 (15/17)	n/a	n/a
**Conjunct., Specificity**	1.0 (33/33)	1.0 (33/33)	n/a	n/a
**COVID-19, Sensitivity**	0.86 (6/7)	0.86 (6/7)	n/a	n/a
**COVID-19, Specificity**	1.0 (43/43)	1.0 (43/43)	n/a	n/a
**Influenza, Sensitivity**	0.62 (5/8)	0.83 (5/6)	n/a	n/a
**Influenza, Specificity**	1.0 (42/42)	1.0 (44/44)	n/a	n/a
**Int. Flu, Sensitivity**	(2/2)	(2/2)	n/a	n/a
**Int. Flu, Specificity**	0.92 (44/48)	0.92 (44/48)	n/a	n/a
**Croup, Sensitivity**	(5/5)	(5/5)	n/a	n/a
**Croup, Specificity**	1.0 (45/45)	1.0 (45/45)	n/a	n/a
**Lice, Sensitivity**	0.88 (7/8)	0.88 (7/8)	n/a	n/a
**Lice, Specificity**	1.0 (42/42)	1.0 (42/42)	n/a	n/a
**Leg Break, Sensitivity**	(1/1)	(1/1)	n/a	n/a
**Leg Break, Specificity**	1.0 (49/49)	1.0 (49/49)	n/a	n/a

Supplement Table S8. - Synthetic posts, Comparative Validations of 6 LLMs vs. GPT-4

LLM Characterizations	Sonnet	Mixtral 8x22b	Opus	LlaMa 3	GPT 4o	GPT 3.5
Outbreak (OB) Probability	0.92 (0.91, 0.93)	0.81 (0.79, 0.83)	0.83 (0.80, 0.86)	0.82 (0.80, 0.84)	0.91 (0.90, 0.92)	0.67 (0.63, 0.71)
OB Severity of Cases	1.0 (0.99, 1.0)	1.0 (0.99, 1.0)	0.99 (0.98, 1.0)	0.99 (0.98, 1.0)	0.99 (0.98, 1.0)	0.98 (0.96, 0.99)
OB Size (# of Cases)	0.81 (0.78, 0.84)	0.75 (0.71, 0.78)	0.79 (0.75, 0.82)	0.80 (0.76, 0.84)	0.75 (0.72, 0.79)	0.71 (0.61, 0.79)
OUTBREAK TYPE						
Infectious, Sensitivity	0.97 (549/565)	0.99 (557/565)	0.98 (554/565)	0.97 (549/565)	0.96 (541/565)	0.99 (562/565)
Infectious, Specificity	0.90 (164/182)	0.92 (168/182)	0.77 (140/182)	0.60 (110/182)	0.95 (173/182)	0.47 (86/182)
Allergic, Sensitivity	0.70 (57/81)	0.90 (73/81)	0.65 (53/81)	0.21 (17/81)	0.86 (70/81)	0.59 (48/81)
Allergic, Specificity	1.0 (666/666)	1.0 (663/666)	1.0 (666/666)	1.0 (666/666)	1.0 (663/666)	1.0 (666/666)
AHC, Sensitivity	0.47 (40/85)	0.85 (72/85)	0.84 (71/85)	0.91 (77/85)	1.0 (85/85)	0.047 (4/85)
AHC, Specificity	1.0 (659/662)	0.99 (654/662)	0.99 (658/662)	0.99 (654/662)	0.96 (637/662)	1.0 (661/662)
Environ., Sensitivity	0.98 (103/105)	0.93 (98/105)	0.92 (97/105)	0.82 (86/105)	0.97 (102/105)	0.33 (35/105)
Environ., Specificity	0.98 (628/642)	0.99 (634/642)	1.0 (642/642)	0.99 (633/642)	1.0 (639/642)	1.0 (641/642)
HEALTH CONDITIONS						
Conjunct., Sensitivity	0.88 (739/837)	0.94 (790/837)	0.98 (822/837)	0.96 (805/837)	0.84 (705/837)	0.87 (726/837)
Conjunct., Specificity	0.62 (194/315)	0.52 (164/315)	0.91 (287/315)	0.49 (155/315)	0.64 (201/315)	0.92 (290/315)
COVID-19, Sensitivity	0.81 (17/21)	0.71 (15/21)	0.86 (18/21)	0.81 (17/21)	0.76 (16/21)	0.52 (11/21)
COVID-19, Specificity	1.0 (1129/1131)	1.0 (1129/1131)	1.0 (1126/1131)	1.0 (1129/1131)	1.0 (1131/1131)	1.0 (1131/1131)
Influenza, Sensitivity	0.90 (43/48)	0.92 (44/48)	0.96 (46/48)	0.92 (44/48)	0.85 (41/48)	0.79 (38/48)
Influenza, Specificity	0.99 (1096/1104)	1.0 (1102/1104)	1.0 (1100/1104)	1.0 (1099/1104)	1.0 (1103/1104)	1.0 (1104/1104)
Int. Flu, Sensitivity	0.52 (34/65)	0.63 (41/65)	0.60 (39/65)	0.55 (36/65)	0.52 (34/65)	0.34 (22/65)
Int. Flu, Specificity	0.99 (1076/1087)	0.98 (1066/1087)	0.98 (1064/1087)	0.99 (1077/1087)	0.99 (1076/1087)	1.0 (1085/1087)
Croup, Sensitivity	0.94 (16/17)	0.76 (13/17)	0.94 (16/17)	0.76 (13/17)	0.76 (13/17)	0.47 (8/17)
Croup, Specificity	1.0 (1133/1135)	1.0 (1134/1135)	1.0 (1133/1135)	1.0 (1133/1135)	1.0 (1134/1135)	1.0 (1135/1135)
Lice, Sensitivity	0.84 (16/19)	0.63 (12/19)	0.84 (16/19)	0.74 (14/19)	0.68 (13/19)	0.16 (3/19)
Lice, Specificity	1.0 (1128/1133)	1.0 (1131/1133)	1.0 (1129/1133)	1.0 (1130/1133)	1.0 (1131/1133)	1.0 (1133/1133)
Leg Break, Sensitivity	0.69 (11/16)	0.62 (10/16)	0.25 (4/16)	0.56 (9/16)	0.56 (9/16)	0.0 (0/16)
Leg Break, Specificity	1.0 (1135/1136)	1.0 (1135/1136)	1.0 (1136/1136)	1.0 (1136/1136)	1.0 (1135/1136)	1.0 (1136/1136)

.
